# Uniquely identifying topological order based on boundary-bulk duality and anyon condensation

**DOI:** 10.1093/nsr/nwac264

**Published:** 2022-11-24

**Authors:** Yong-Ju Hai, Ze Zhang, Hao Zheng, Liang Kong, Jiansheng Wu, Dapeng Yu

**Affiliations:** Shenzhen Institute for Quantum Science and Engineering, Southern University of Science and Technology, Shenzhen 518055, China; Department of Physics, Southern University of Science and Technology, Shenzhen 518055, China; Department of Physics, Southern University of Science and Technology, Shenzhen 518055, China; Shenzhen Institute for Quantum Science and Engineering, Southern University of Science and Technology, Shenzhen 518055, China; International Quantum Academy, Shenzhen 518048, China; Guangdong Provincial Key Laboratory of Quantum Science and Engineering, Shenzhen 518055, China; Shenzhen Institute for Quantum Science and Engineering, Southern University of Science and Technology, Shenzhen 518055, China; International Quantum Academy, Shenzhen 518048, China; Guangdong Provincial Key Laboratory of Quantum Science and Engineering, Shenzhen 518055, China; Shenzhen Institute for Quantum Science and Engineering, Southern University of Science and Technology, Shenzhen 518055, China; International Quantum Academy, Shenzhen 518048, China; Guangdong Provincial Key Laboratory of Quantum Science and Engineering, Shenzhen 518055, China; Shenzhen Institute for Quantum Science and Engineering, Southern University of Science and Technology, Shenzhen 518055, China; Department of Physics, Southern University of Science and Technology, Shenzhen 518055, China; International Quantum Academy, Shenzhen 518048, China; Guangdong Provincial Key Laboratory of Quantum Science and Engineering, Shenzhen 518055, China

**Keywords:** topological order, *R* matrix, *F* matrix, bulk-boundary duality, anyon condensation

## Abstract

Topological order is a new quantum phase that is beyond Landau’s symmetry-breaking paradigm. Its defining features include robust degenerate ground states, long-range entanglement and anyons. It was known that *R* and *F* matrices, which characterize the fusion-braiding properties of anyons, can be used to uniquely identify topological order. In this article, we explore an essential question: how can the *R* and *F* matrices be experimentally measured? We show that the braidings, i.e. the *R* matrices, can be completely determined by the half braidings of boundary excitations due to the boundary-bulk duality and the anyon condensation. The *F* matrices can also be measured by comparing the quantum states involving the fusion of three anyons in two different orders. Thus we provide a model-independent experimental protocol to uniquely identify topological order. By using quantum simulations based on a toric code model with boundaries encoded in three- and four-qubit systems and state-of-the-art technology, we obtain the first experimental measurement of *R* and *F* matrices by means of an NMR quantum computer at room temperature.

## Teaser

A protocol to uniquely identify topological orders by measuring the fingerprints of anyons is proposed and demonstrated in few-qubit systems.

## INTRODUCTION

Topological orders are defined as gapped many-body systems at zero temperature. They were first discovered in the two-dimensional (2D) fractional quantum Hall effect (FQHE), and are new types of quantum phases beyond Landau’s symmetry-breaking paradigm [1–17]. Not only do they challenge us to find a radically new understanding of phases and phase transitions, but also provide the physical foundation of fault-tolerant topological quantum computers (TQCs) [18–22].

A fundamental question is how to characterize and measure a topological order precisely. The key features of topological order in the FQHE are fractional charges and fractional statistics. The detection of them has been a major direction of recent research [23]. The related experiments in realistic systems are mainly about interferometry [24–28], anyon collider [29–31] and transport experiments [32–36]. Because of the subtle effect of Coulomb interaction and the participation of other quasiparticles, these experiments are very challenging and their interpretation is complicated [23]. On the other hand, in artificial quantum processors, the approach of quantum simulation has been highly developed to study these topological orders due to their controllability and measurability [37]. Important progress has been made toward achieving this goal, such as the measurement of modular data, i.e. *S* matrices and *T* matrices in a few-qubit system and the measurement of topological entanglement entropy [6,7,38–45]. This motivated a folklore belief among experts that the modular data might be complete [46]. A recent mathematical result [47], however, shows that it is incomplete, in the sense that different topological orders might have the same modular data. The complete characterization of topological order should be *R* and *F* matrices.

**Figure 1.**
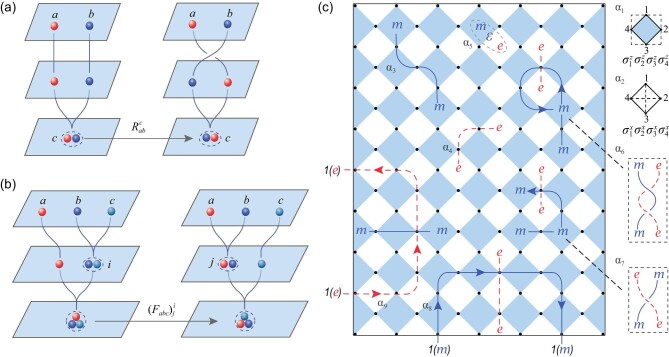
Graphical representations of *R* matrices, *F* matrices and a toric code lattice with gapped boundaries. (a) Braiding of two anyons and the definition of *R* matrices. (b) Fusion of three anyons in different orders and the definition of *F* matrices. (c) Toric code lattice with boundaries. Here α_1_ and α_2_ are two elementary plaquettes: the blue plaquette and the white plaquette; α_3_ and α_4_ are the string operators for generating an *m* anyon pair and an *e* anyon pair, respectively; α_5_ shows the fusion of an *m* and an *e* to produce a new ϵ anyon. The string operators of the two anyons are omitted for clarity. The double braiding and braiding operations are visualized with α_6_ and α_7_. Double braiding corresponds to moving an *m* (*e*) anyon around an *e* (*m*) anyon along a full circle, which generates an overall phase of −1. Braiding corresponds to the exchange of an *m* anyon and an *e* anyon: if they are in the bulk of the lattice model, the state after braiding differs from the initial state not only by a phase factor. α_8_ and α_9_ are half braidings at the white and blue boundaries. At a white boundary, an *m* anyon is equivalent to vacuum state 1. Dragging an *m* anyon from the vacuum state, moving it around a boundary excitation *e* along a semicircle and pushing it back out results in a phase difference of −1. The case is similar for a blue boundary.

A 2D topological order permits particle-like topological excitations, called anyons. Two anyons can be fused together to produce a new anyon, from one or several possible outcomes. Consider the case that anyons *a* and *b* are fused to produce anyon *c*, we compare two processes. One is to fuse two anyons *a* and *b* to produce anyon *c* directly, and the other process is to braid (move one anyon around another along a semicircle, i.e. exchange) these two anyons first, then fuse them to produce anyon *c*. These two processes end up with the same anyon *c* if *a* and *b* are within a distance smaller than the correlation length of the ground state of the system [48]. In this case, the inner structure of anyon *c*, i.e. the relative positions of anyons *a* and *b* can be ignored; thus, their wave functions can only be different by a ‘phase factor’ (or a matrix). This is the so-called *R* matrix, as illustrated in Fig. 1(a). Furthermore, one can fuse three anyons *a, b* and *c* in two different ways: ((*ab*)*c*) and (*a*(*bc*)) with parentheses indicating the order of the fusion. The first set of fused states spans the same Hilbert space as the second one and the *F* matrix is the transformation matrix between these two bases, as illustrated in Fig. 1(b). Mathematically, it was proven that *R* matrices and *F* matrices uniquely determine the topological order [4,5]. In addition to this, *R* matrices, i.e. the braidings, are the fundamental operations in TQCs [18–22].

Therefore, the essential question is whether *R* matrices (braidings) and *F* matrices are physically measurable. The difficulty in measuring *R* matrices is two-fold. First, different gauge choices of *R* matrices might define the same topological order, so they are not unique and seem not to be measurable quantities. Second, by the definition of braiding, if we move one anyon along a semicircle around another anyon of a different kind in the bulk, the spatial configuration of the final state is different from the initial one such that there is no well-defined phase factor. In continuous models, such a difference in spatial configurations, i.e. the information of the relative positions of these two anyons, can be erased by putting them in a region smaller than the correlation length. However, this procedure does not work for lattice models since in most lattice models of topological order, two anyons cannot be put into one lattice site.

In this paper, we address this fundamental question and overcome these two difficulties using the theories of boundary-bulk duality [49,50] and anyon condensation on the boundaries [22,51–53]. The theories show that the braidings among bulk anyons are determined by the half braidings among boundary excitations, and a half braiding is defined and measured by moving one boundary excitation around another one along a semicircle near the boundary. We find different but gauge-equivalent *R* matrices are associated with different boundaries of the same 2D topological order. When the boundary is gapped, certain bulk anyons are condensed on the boundary, and thus can be created or annihilated on the boundary by local operators.

By creating an anyon at the boundary, then half braiding it with another anyon and then annihilating it on the boundary, we obtain a final state that differs from the initial state only by a phase factor (*R* matrix) and overcome the second difficulty. We also show that the *F* matrices are measurable using a quantum circuit involving the fusion of three anyons in different orders. As a consequence, we provide a protocol for experimentally measuring the *R* matrices and *F* matrices. We demonstrate our protocol through quantum simulation of a few-qubit toric code model with gapped boundaries, where we mainly focus on the *R* matrices measurement and present a proof-of-principle measurement of the trivial *F* matrices.

## BOUNDARY-BULK DUALITY AND ANYON CONDENSATIONS

In this section, we explain the boundary-bulk duality and anyon condensations that enable the measurement of *R* matrices in the context of the toric code model. As the simplest example of the *Z*_2_ topological order, the toric code model is a useful platform for demonstrating anyonic statistics [18]. It is defined on a 2D square lattice consisting of two kinds of plaquettes with qubits on their edges, as illustrated in Fig. 1(c).

The toric code Hamiltonian is a sum of all four qubit interaction terms for the plaquettes (stabilizer operators) in the lattice:


(1) \begin{eqnarray*} H = - \sum _{\text{white plaquettes}} A_p - \sum _{\text{blue plaquettes}} B_p. \end{eqnarray*}

The operators $A_p=\Pi _{j\in \partial p} \sigma ^x_j$ and $B_p=\Pi _{j\in \partial p} \sigma ^z_j$ are the plaquette operators acting on the four qubits surrounding white plaquettes and blue plaquettes, respectively, as shown by α_1_ and α_2_ in Fig. 1(c). Here $\sigma ^x_j$ and $\sigma ^z_j$ are the *x* and *z* components of Pauli matrices, respectively, acting on the *j*-th of the four qubits for each plaquette. All of the above plaquette operators commute with each other, and their eigenvalues are ±1. The ground state (or vacuum, denoted by 1) of the system is the state in which all of the plaquette operators are in +1 eigenstates. Particle-like excitations (anyons) can be created in pairs via string operators. As shown by α_3_ and α_4_ in Fig. 1(c), the blue (red) string operator consists of a sequence of $\sigma ^x_j$ ($\sigma ^z_j$) operators acting on all qubits on the string. Since the string operator anticommutes with a pair of plaquette operators at its end, when the blue (red) string operator is applied to the ground state, the resulting excited state is in the −1 eigenstate of the plaquette operators at the two ends of the string, leading to the creation of a pair of particle-like excitations called *m* anyons (*e* anyons) on the blue (white) plaquettes at the two ends of the string. The combination (fusion, denoted as ‘⊗’) of anyons can produce new types of anyons. Because of the fact that *m* and *e* anyons can be created in pairs, they are their own antiparticles and the fusion of two *m* (or *e*) anyons gives the vacuum, while the fusion of an *e* and an *m* forms a new anyon denoted ϵ, as shown by α_5_ in Fig. 1(c). We obtain the fusion rules: *e* ⊗ *e* = *m* ⊗ *m* = 1 and *e* ⊗ *m* = ϵ.

Anyons can be braided. As illustrated by α_6_ in Fig. 1(c), when an *m* anyon is moved around an *e* anyon along a full circle (double braiding), it produces an overall phase of −1 between the final and initial states. This phase is encoded in the *S* matrices. Another important type of data is the topological spins of the anyons encoded in *T* matrices. The *S* and *T* matrices were believed by many to characterize a topological order uniquely, and, in the case of the toric code, can be measured by experiments [18,38,41]. However, as mentioned in the Introduction, *S* and *T* matrices were proven to be inadequate to uniquely identify a topological order [47]. For unique identifications, we need *R* matrices (braidings) and *F* matrices. The *F* matrices are trivial (factors of 1) for the toric code model. The *R* matrices are actually phase factors for the anyons in the toric code model since the anyons here are Abelian. Two typical *R* matrices for the toric code are $R_{me}^{\varepsilon }=-1$ and $R_{em}^{\varepsilon }=+1$. The measurement of *R* matrices is the main focus of this article.

**Figure 2.**
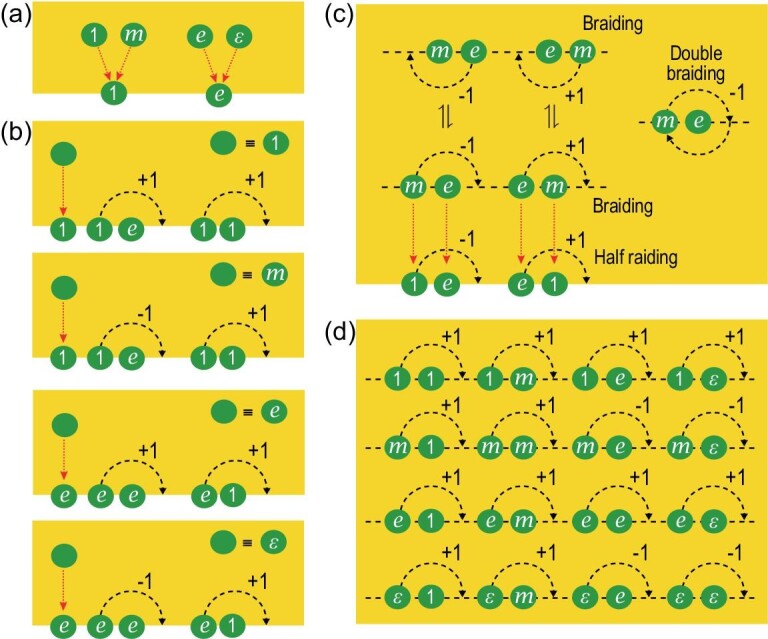
Illustrations of half braidings, braidings and double braidings. (a) There are four kinds of bulk anyons in the bulk and two kinds of boundary excitations. Anyons *m* and 1 (*e* and ϵ) belong to the same topological sector when they are moved to a white boundary. (b) Definitions of the four kinds of bulk anyons using the half braidings on the boundary. Anyons *m* and 1 are different when they are equipped with different half braidings, as are ϵ and *e*. (c) Braidings in the bulk defined in terms of the half braidings on the boundary. The black dotted line represents a virtual boundary in the bulk. By moving bulk anyons to the boundary, and using the half braidings of the boundary excitations, the braidings of the bulk anyons can be defined. There are two equivalent sets of braidings (the set above the virtual boundary and the set below it) and the double braidings can be obtained from either set of these braidings as well. (d) A complete list of braidings for the four kinds of bulk anyons.

The key to measuring the braidings (*R* matrices) is to make use of the boundaries due to the boundary-bulk duality [49,50] and the anyon condensation on the boundaries [22,51–53]. There are two topologically distinct boundary types in the toric code [22], namely the white boundaries (known as the smooth boundaries in the original vertex-plaquette version of the toric code model) and the blue boundaries (rough boundaries in the original version), as shown in Fig. 1(c). When an *m* anyon approaches a white boundary, it disappears completely or condenses to the vacuum. This means that the *m* anyon condenses on the boundary. Here, the meaning of ‘anyon condensation’ is that a single anyon can be annihilated or created by local operators on the boundary. As we know, local operators do not change the topological sectors of states. So, by local operators, an anyon can only be created with its antiparticle from the vacuum. The usual way to create a single anyon is to perform a series of local operators, i.e. string operators, to separate it from its antiparticle and push the antiparticle to infinite. But due to the existence of boundaries, the condensed particles (i.e. *m* anyon) can be created by local operators on the boundaries. However, an *e* anyon cannot pass and becomes a boundary excitation. Therefore, the boundary excitations on a white boundary are {1, *e*}, and the bulk anyons map to the boundary excitations as 1, *m* ↦ 1, *e*, ϵ ↦ *e* (as illustrated in Fig. 2(a)). At a blue boundary, *e* anyons disappear and *m* anyons remain. Therefore, the blue boundary excitations are {1, *m*} and the corresponding bulk-to-boundary map is 1, *e* ↦ 1, *m*, ϵ ↦ *m*. Clearly, the bulk-boundary map is not one to one.

It turns out that bulk anyons can be uniquely determined by the excitations at either of these boundaries [49–51]. For example, consider a white boundary where *m* anyons condense. On this boundary, *m* and 1 belong to the same topological sector, i.e. *m* = 1. However, when an *m* is moved into the bulk, it is automatically endowed with additional structures called *half braidings*. These half braidings can be measured by moving the *m* around a 1 or *e* along a semicircle near the boundary, as illustrated by α_8_ in Fig. 1(c). It is easy to check that moving an *m* around a 1 along a semicircle does not result in a phase difference, whereas moving an *m* around an *e* along a semicircle results in a phase difference of −1. Consequently, an *m* anyon in the bulk can be completely characterized by the triple


(2) \begin{eqnarray*} m= (1,1\otimes 1 &=&1\stackrel{1}{\rightarrow }1=1\otimes 1,\\ 1\otimes e &=& e\stackrel{-1}{\rightarrow } e=e\otimes 1), \end{eqnarray*}

where the first component means that the *m* = 1 on the boundary and the second and third components are half braidings, which are physically measurable quantities. Therefore, bulk anyons are precisely boundary excitations equipped with half braidings. In this way, we can recover all four bulk anyons in the forms of four triples, as illustrated in the top panel of Table 1 and Fig. 2(b).

**Table 1.** The bulk anyons defined by boundary excitations and the bulk anyon braidings defined by the half braidings of boundary excitations.

The bulk anyons defined by the boundary excitations and half braidings
$1=(1,1\otimes 1=1\stackrel{1}{\rightarrow } 1=1\otimes 1,1\otimes e= e\stackrel{1}{\rightarrow } e=e\otimes 1)$
$m= (1,1\otimes 1=1\stackrel{1}{\rightarrow } 1=1\otimes 1,1\otimes e =e \stackrel{-1}{\rightarrow } e=e\otimes 1)$
$e= (e,e\otimes 1=e\stackrel{1}{\rightarrow } e=1\otimes e, e\otimes e=1\stackrel{1}{\rightarrow } 1=e\otimes e)$
$\varepsilon = (e,e\otimes 1=e \stackrel{1}{\rightarrow } e=1\otimes e,e\otimes e=1 \stackrel{-1}{\rightarrow } 1=e\otimes e)$
The bulk anyon braidings defined by the half braidings of boundary excitations
$1 \otimes x = x \stackrel{c_{1, x}=1}{\longrightarrow} x = x\otimes 1$ , for *x* = 1, *e, m*, ϵ
$x\otimes x = 1 \stackrel{c_{x, x}=1}{\longrightarrow} 1 = x\otimes x$ , for *x* = 1, *e, m*
$\varepsilon \otimes \varepsilon = 1 \stackrel{c_{\varepsilon , \varepsilon }= -1}{\longrightarrow} 1 = \varepsilon \otimes \varepsilon$
$e\otimes m = \varepsilon \stackrel{c_{e,m}=1}{\longrightarrow} \varepsilon = m\otimes e$
$m\otimes e = \varepsilon \stackrel{c_{m,e}=-1}{\longrightarrow} \varepsilon = e\otimes m$
$e \otimes \varepsilon =m \stackrel{c_{e,\varepsilon }=1}{\longrightarrow} m=\varepsilon \otimes e$
$\varepsilon \otimes e =m \stackrel{c_{\varepsilon ,e}=-1}{\longrightarrow} m= e \otimes \varepsilon$
$m\otimes \varepsilon =e \stackrel{c_{m,\varepsilon }=-1}{\longrightarrow} e= \varepsilon \otimes m$
$\varepsilon \otimes m =e \stackrel{c_{\varepsilon ,m}=1}{\longrightarrow} e= m\otimes \varepsilon$

Furthermore, the braidings among these four anyons can be defined by the half braidings [54]. For example, we can obtain the braiding


(3) \begin{eqnarray*} m\otimes e = 1\otimes e \stackrel{-1}{\rightarrow } e\otimes 1 = e \otimes m. \end{eqnarray*}

Here we have used the fact that *m* = 1 on the boundary and the half braiding −1 on the boundary, as illustrated in Fig. 2(c). Thus, we obtain $R_{me}^{\varepsilon }=-1$. In addition, since *m* condenses (*m* = 1) on the boundary, $R_{em}^{\varepsilon }=1$ (For $R_{bm}^c$, *b* is the moving anyon and *m* is the anyon fixed on the boundary. Since *m* = 1 on the boundary, $R_{bm}^{c}=1$ for an arbitrary *b* anyon. For $R_{mb}^{c}$, *b* is the fixed anyon on the boundary, and *m* is the moving anyon. A nontrivial half braiding is applied in this case.) All of the braidings of bulk anyons can be obtained in this way, as listed in the bottom panel of Table 1 and illustrated in Fig. 2(d). Thus, we can obtain the bulk braidings from the boundary half braidings, which are measurable. Note that a one-to-one mapping from the bulk braidings to boundary half braidings can be obtained by considering a virtual boundary in the bulk, as illustrated in Fig. 2(c). Since the bulk braidings are defined by the boundary half braidings, which are physically measurable, the bulk braidings are also physically measurable. Similarly, the same four anyons {1, *e, m*, ϵ} in the bulk can be reconstructed from excitations {1, *m*} on the blue boundary by again defining four triples from a new set of half braidings, which further defines in the bulk a new set of braidings that is different but equivalent to that constructed from the white boundary. Therefore, a boundary condition of the model provides us with a physical way to fix the gauge freedoms of the braidings. Double braidings are independent of the gauge and can be obtained from either set of boundary half braidings, as shown in Fig. 2(c), and are explained in detail in the online supplementary material.

In a formal mathematical language, excitations {1, *e*} on the white boundary form a mathematical structure called a unitary fusion category (UFC), denoted ${\rm Rep}({\mathbb {Z}}_2)$, i.e. ${\rm Rep}({\mathbb {Z}}_2)=\lbrace 1, e\rbrace$. Our construction of the four triples in the top panel of Table 1 precisely repeats in a completely physical way the mathematical definition of the Drinfeld center of ${\rm Rep}({\mathbb {Z}}_2)$. This Drinfeld center, denoted ${\rm Z}({\rm Rep}({\mathbb {Z}}_2))$, is precisely the unitary modular tensor category (UMTC) of the bulk anyons, i.e. ${\rm Z}({\rm Rep}({\mathbb {Z}}_2))=\lbrace 1, e, m, \varepsilon \rbrace$. In other words, we obtain a physical proof of the boundary-bulk duality [49,50]. Similarly, the excitations on the blue boundary form a UFC ${\rm Vec}_{{\mathbb {Z}}_2}$. Its Drinfeld center ${\rm Z}({\rm Vec}_{{\mathbb {Z}}_2})$ again reproduces all the bulk anyons. Mathematically, ${\rm Z}({\rm Rep}({\mathbb {Z}}_2))$ and ${\rm Z}({\rm Vec}_{{\mathbb {Z}}_2})$ are equivalent as UMTCs. This equivalence is the source of the gauge freedoms of the braidings. Note that the toric code model is a special case of the quantum double model (QDM) with group $\mathbb {Z}_2$. A general measurement protocol of anyon braiding based on the bulk-boundary duality for the QDM can be found in the online supplementary material.

## MEASUREMENT OF THE *R* MATRICES

We present our experimental demonstration of the measurement of *R* and *F* matrices for the toric code model in the following sections.

An ideal quantum simulation should be a simulation of a quantum system of which the Hamiltonian is the toric code model and we do the braidings and measure the *R* and *F* matrices. It is challenging since the four-body interaction is hard to achieve. Fortunately, information about the topological order, i.e. information about anyons, is completely contained in the wave functions of the system [39]. The Hamiltonian is important when we consider fault-tolerated TQCs using anyons since it is the gap induced by the Hamiltonian that makes the information stored in the topological states robust. To get the braiding-fusion properties of anyons, wave functions of the system are enough, which is the main focus of our simulation.

**Figure 3.**
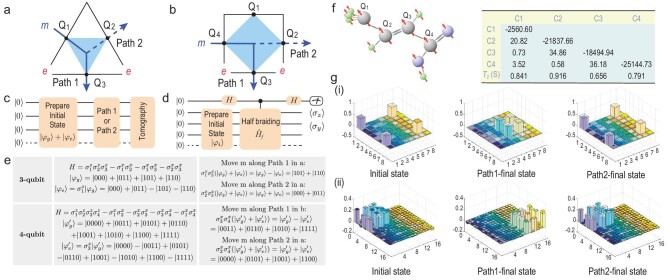
Illustrations of the experimental setups and corresponding quantum states, the experimental platforms and the results of quantum state tomography. (a) A three-qubit toric code model and the half braidings of *m* along path 1 and path 2. (b) A four-qubit toric code model and the half braidings of *m* along path 1 and path 2. (c) Quantum circuit for measuring *m*-*e* half braidings on a white boundary. The initial state |ϕ_g_〉 + |ϕ_e_〉 is prepared at first, where |ϕ_g_〉 is the ground state and |ϕ_e_〉 represents the excited state with two boundary *e* excitations. Moving the *m* anyon through path 1 and path 2 by applying a series of σ_*x*_ operators to the qubits involved in these paths leads to states |ϕ_g_〉 − |ϕ_e_〉 and |ϕ_g_〉 + |ϕ_e_〉, respectively. These two states can be differentiated via quantum state tomography. (d) Quantum circuit for the general phase measurement. The state before half braiding is prepared as the initial state |ϕ_*i*_〉, and a half braiding is performed as a controlled operation. The phase factor induced by half braiding can be obtained from the two expectation values 〈σ_*z*_〉 and 〈σ_*y*_〉 on the ancilla qubit. (e) The Hamiltonians and quantum states in the experimental processes for the three- and four-qubits systems. (f) Our three-, four-qubit quantum simulator is a sample of ^13^C-labeled trans-crotonic acid molecules. We make four ^13^C atoms from the sample as four qubits. The table on the right lists the parameters of the chemical shifts (diagonal, hertz ), J-coupling strengths (off-diagonal, hertz ) and relaxation time scales *T*_2_ (seconds). (g(i)) State tomography results for the initial and final states obtained when moving *m* along different paths in the three-qubit toric code model. The transparent columns represent the theoretical values, and the colored columns represent the experimental results. Regarding the tick labels of the horizontal axes in each three-dimensional bar graph, 1 represents state |0000〉, 2 represents state |0001〉 and so on. Compared with the theoretical results, the two final states in the three-qubit experiments using path 1 and path 2 are obtained with fidelities 96.37% and 96.67%, respectively. (g(ii)) State tomography results for the final states obtained by moving *m* along different paths in the four-qubit toric code model. Compared with the theoretical results, the two final states in the four-qubit experiments using path 1 and path 2 are obtained with fidelities 95.23% and 95.21%, respectively.

We mainly focus on the measurement of $R_{me}^{\varepsilon }=-1$ since it is the most important nontrivial phase factor in the toric code. Our measurement is performed in three- and four-qubit models with white boundaries. The half braiding can be implemented by creating a condensed *m* anyon at the boundary, moving it around a boundary excitation *e* along a semicircle and annihilating it on the boundary. To measure the phase factor induced by the half braiding, we make use of either a superposition of the ground state and excited state or a scattering circuit. The experimental setup and quantum circuits are illustrated in Fig. 3(a)–(c), and the specific quantum states involved can be seen in Fig. 3(e).

For the three-qubit case (Fig. 3(a)), the Hamiltonian and the ground state |ϕ_g_〉 of the triangular cell are shown in Fig. 3(e). The excited state |ϕ_e_〉 can be obtained via a σ_*z*_ rotation of qubit 1 in the ground state, leading to two *e* anyons on the lower two vertices. In this system, two different braiding processes can be performed by moving *m* along either path 1 or path 2. Braiding along path 1 results in overall phase factors of +1 for the ground state and −1 for the excited state since in the latter case a half braiding of *m* around *e* is performed. In contrast, path 2 is trivial and it does not generate any phase factor difference. To measure the phase factor difference, we prepare an initial state that is a superposition of the ground state and excited state, |ϕ_g_〉 + |ϕ_e_〉. Then, half braidings along path 1 and path 2 give rise to the final states |ϕ_g_〉 − |ϕ_e_〉 and |ϕ_g_〉 + |ϕ_e_〉, respectively, which can be identified by quantum state tomography. The four-qubit case is similar, as shown in Fig. 3(b) and (e). The corresponding quantum circuits for this experiment are shown in Fig. 3(c).

In Fig. 3(g), we present the state tomography results obtained after running these quantum circuits on our NMR qubit platform. After moving *m* along path 2, we obtain a final state that is the same as the initial state. In contrast, after moving *m* along path 1, we obtain a final state that is completely different from the initial state, which is due to the phase factor of −1 induced by the half braiding of *m* around *e*. The average state fidelities after path 1 and path 2 are 96.37$\%$ and 96.67$\%$ for the three-qubit system and 95.23$\%$ and 95.21$\%$ for the four-qubit system. Thus we obtain the braiding in the form of *R* matrices, $R_{me}^{\varepsilon }=-1$. In principle, all other braidings can be similarly obtained.

In general, a half braiding (denoted $\hat{H}_f$) leads to a phase factor for Abelian anyons, which can be measured by means of a scattering circuit with one additional ancilla control qubit [41,55], as shown in Fig. 3(d). The state before half braiding is prepared as the initial state |ϕ_*i*_〉, and the half braiding is performed as a controlled operation. In our experiment, the state before half braiding is prepared as the initial state |ϕ_*i*_〉 with fidelity 95.32%. The global phase generated by half braiding is obtained from the two expectation values 〈σ_*z*_〉 and 〈σ_*y*_〉 on the ancilla qubit through the relations $\langle {{\sigma _z}} \rangle = {\mathop {\rm Re}\nolimits } (\langle {{\varphi _i}} |\hat{H}_f | {{\varphi _i}} \rangle )$ and $\langle {{\sigma _y}} \rangle = {\mathop {\rm Im}\nolimits } (\langle {{\varphi _i}} |\hat{H}_f | {{\varphi _i}} \rangle )$. This method can also be applied to the case of the non-Abelian anyon, in which the measured values are the matrix elements of the *R* matrices. This circuit is tested for *m*-*e* half braiding on a three-qubit plaquette on NMR qubits as a proof of principle. Here $R^{\varepsilon }_{me}=-1$ theoretically corresponds to an exchange statistics with a phase of π, and we obtain values of (1.027 ± 0.001)π in the experiment.

## MEASUREMENT OF THE *F* MATRICES

**Figure 4.**
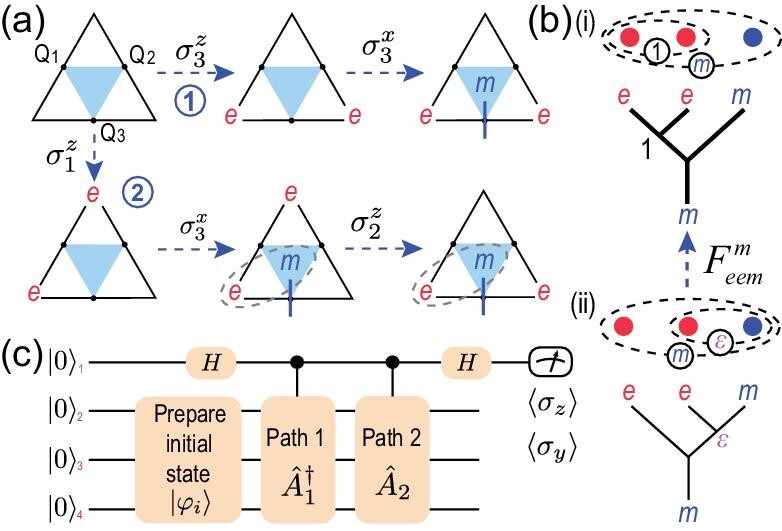
Two fusion diagrams and measurement of the *F* matrix. (a) Two different fusion processes on a three-qubit plaquette represented by two paths. (b) The circle notation and fusion tree diagrams for path 1 and path 2 in (a). (c) The scattering circuit used to measure the overlap of the final states of the two paths, where $\hat{A}_1 = \sigma _3^x \sigma _3^z$ and $\hat{A}_2 = \sigma _2^z \sigma _3^x \sigma _1^z$. The value of the overlap yields $F_{eem}^{m}$.

The *F* matrices can be measured by means of a similar scattering circuit [41,55] shown in Fig. 4(c). We demonstrate the measurement of a typical matrix $F_{eem}^m$ as follows.

First, the ground state without any anyons is prepared as the initial state |ϕ_*i*_〉 before fusion, then two controlled operations $\hat{A}_{1,2}$ are applied, which represent two fusion processes using different fusion orders to fuse two *e* anyons and an *m* into an *m* anyon, as illustrated in Fig. 4(b). The global phase generated by the different fusion orders, $F_{eem}^m$, is obtained from the two expectation values 〈σ_*z*_〉 and 〈σ_*y*_〉 of the ancilla qubit.

We experimentally measure $\langle {{\sigma _4 ^z}} \rangle$ and $\langle {{\sigma _4 ^y}} \rangle$ and obtain $\langle {{\sigma _4 ^z}} \rangle = 0.712\pm 0.006$ and $\langle {{\sigma _4 ^y}} \rangle = 0.177\pm 0.004$. We normalize them such that their squares sum to 1 and obtain the angle $\theta =\arctan ({\langle {{\sigma _4 ^y}} \rangle }/{\langle {{\sigma _4 ^z}} \rangle }) =(0.077\pm 0.002)\pi$, which is close to its theoretical value 0. Thus, we verify that $F_{eem}^{m}=1$ in this experiment.

## DISCUSSION

In summary, we experimentally measure anyon braidings (*R* matrices) through the boundary-bulk duality—bulk anyons are those boundary excitations equipped with half braidings, and bulk anyon braidings can be obtained from boundary excitation half braidings. Two difficulties that arise in the measurement of *R* matrices, as noted in the Introduction, can be overcome by means of the boundary-bulk duality and the anyon condensation on the boundaries: (1) if we instead consider the blue boundary, where *e* anyons condense, we obtain another set of *R* matrices that is gauge equivalent to what is measured here, and (2) if an anyon is created on the boundary, half braided with another anyon, and then annihilated on the boundary, the final state and initial state differ only by a phase factor. These two difficulties seem to be technical problems, but they are actually related to the following essential question: what are the fundamental quantities that characterize topological order? This question is similar to the following one: which is the fundamental quantity for an electromagnetic field, the magnetic/electric field or the vector/scaler potential? It would seem that, since the vector potential is a gauge-dependent quantity, which means that it is not unique, it should not be measurable. However, the Aharonov-Bohm effect shows that, when a particle passes through a region where the magnetic field is zero but the vector potential is nonzero, the phase of the wave function is shifted [56]. This proves that the vector potential, rather than the magnetic field, is the fundamental physical quantity. The relation between the double braiding and braiding of anyons is similar to that between the magnetic field and the vector potential. For a double braiding of two anyons, the spatial configuration of the final state is the same as that of the initial state, so the effect of the anyonic statistics can be measured by comparing the phase (it is a unitary matrix for non-Abelian anyons) before and after the double braiding. But for a braiding, i.e. an exchange in positions of two anyons, the spatial configuration of the final state is different from that of the initial state if these two anyons are of different kinds, which makes it unmeasurable in bulk for the lattice model (for a continuous model, such a spatial configuration difference can be ignored when two anyons are within a region smaller than the correlation length of the ground state). Furthermore, the *R* matrices (braidings) are not unique for a given topological order. Therefore, it would seem that double braidings should be the fundamental quantities for topological orders. However, our experiments have demonstrated the important fact that braidings, rather than double braidings, are the fundamental physical quantities for topological orders.

The *F* matrices can also be measured using a scattering quantum circuit involving the fusion of three anyons in different orders. The *S* and *T* matrices can be calculated from the *R* the *F* matrices [4,5]. Thus, we provide an experimental protocol for uniquely identifying topological orders. Although our results are obtained on only a few qubits, the conclusion is applicable to large systems since the toric code is at a fixed point and the conclusion is independent of the system size [57]. Furthermore, our boundary-bulk duality between bulk anyons and boundary excitations and the correspondence between bulk anyon braidings and boundary excitation half braidings also holds for other topological orders, even for non-Abelian anyons such as Fibonacci anyons, semions and the QDM of the *S*_3_ group [50]. This protocol is model independent. The idea of measuring the half braidings should also be useful to the experimental study of gapless boundaries [58,59]. For a 2D topological order with chiral gapless boundaries, one can apply the folding trick to embed the measurability problem to that of a double-layered system with only gapped boundaries.

Given the special role of anyon braidings and topological orders in strongly correlated systems and TQCs, our work has potential applications not only in uniquely identifying these exotic phases of matter but also in describing the interplay of bulk physics and boundary physics in topological systems. The recently proposed surface code, using the toric code with boundary and geometric defects, provides a scheme for practical large-scale quantum computation [60]. The existence of boundaries and defects provides myriad possibilities for the manipulation of topological quantum states [61]. A system with boundaries where anyons can condense is proposed to construct universal TQCs [62]. It will be interesting to consider a TQC protocol in which new operations, half braidings of boundary excitations, are introduced. Such a system can perhaps be used to construct universal TQCs with simpler quantum gates.

## MATERIALS AND METHODS

Our experiments are accomplished by means of a four-qubit NMR quantum processor. The system is a sample of ^13^C-labeled trans-crotonic acid molecules dissolved in d6-acetone. The sample consists of four ^13^C atoms, as shown in Fig. 3(f), and all experiments are conducted on a Bruker Ascend NMR 600 MHz spectrometer at room temperature. In the second and third experiments, we choose Q3 in the molecule (Fig. 3(a)) as the control qubit. Qubits Q1, Q2 and Q4 in the molecule represent Q1, Q2 and Q3 in the circuit, respectively.

In the first experiment, our goal is to show an experimental proof-of-principle demonstration of the half braidings on a gapped boundary and show the effect of the nontrivial phase factor induced by it. We create a condensed *m* anyon at the boundary and move it around a boundary excitation *e* along a semicircle. This is the most important nontrivial half braiding in the toric code. The experimental setup and quantum circuits are illustrated in Fig. 3(a)–(c), and the specific quantum states involved can be seen in Fig. 3(e). The experiment can be divided into three steps: (1) prepare the initial state, i.e. a superposition of the ground state and the excited state; (2) perform half braiding and a trivial braiding by a series of single-qubit rotation operators, which corresponds to moving anyons through path 1 and path 2 in Fig. 3(a) and (b), respectively; and (3) measure the final state and use quantum state tomography to obtain the density matrix of the final state. For path 1, we obtain a final state that is different from the initial state due to the nontrivial phase factor induced by *m*-*e* half braiding. For path 2, we obtain a final state that is the same as the initial one.

In the second experiment, to directly measure the phase factor induced by half braiding, an ancilla qubit is introduced. The state before half braiding is prepared as the initial state |ϕ_*i*_〉 and the half braiding is performed as a controlled operation, as shown in Fig. 3(d). Finally, the two expectation values 〈σ_*z*_〉 and 〈σ_*y*_〉 on the ancilla qubit are measured to obtain the real and imaginary parts of the phase factor generated by half braiding from $\langle {{\sigma _z}} \rangle = {\mathop {\rm Re}\nolimits } (\langle {{\varphi _i}} |\hat{H}_f | {{\varphi _i}} \rangle )$ and $\langle {{\sigma _y}} \rangle = {\mathop {\rm Im}\nolimits } (\langle {{\varphi _i}} |\hat{H}_f | {{\varphi _i}} \rangle )$. The measurement of *m*-*e* half braiding on a three-qubit plaquette is performed using this circuit.

The third experiment is to measure *F*-matrix $F_{eem}^{m}$. The ground state |ψ_*g*_〉 of the three-qubit toric code model is prepared with fidelity $92.96\%$. Then, the operators $\hat{A}_1^\dagger = (\sigma _3^x \sigma _3^z)^\dagger$ and $\hat{A}_2=\sigma _2^z \sigma _3^x \sigma _1^z$ (corresponding to path 1 and path 2 in Fig. 4(a)), which represent two different orders of fusion of three anyons, are applied to the three-qubit toric code under the control of qubit Q4. We measure $\langle {{\sigma _4 ^z}} \rangle$ and $\langle {{\sigma _4 ^y}} \rangle$ of the control qubit to obtain the overlap of the two final states from two paths; thus, obtain the angle $\theta =\arctan ({\langle {{\sigma _4 ^y}} \rangle }/{\langle {{\sigma _4 ^z}} \rangle })$ and $F_{eem}^{m}=\exp (i\theta )$.

Detailed theory and detailed descriptions of the experimental processes and data are provided in the online supplementary material.

## Supplementary Material

nwac264_Supplemental_File
