# Supplementary material for: Uniquely identifying topological order based on boundary-bulk duality and anyon condensation
Source: Natl Sci Rev. 2022 Nov 24;10(3):nwac264. doi: 10.1093/nsr/nwac264 (PMC10007699; doi:10.1093/nsr/nwac264)
Supplement: nwac264_Supplemental_File [file nwac264_supplemental_file.pdf]

Supplementary Material for "Uniquely Identifying Topological Order Based on Boundary-Bulk Duality and Anyon Condensation"

Yong-Ju Hai, Ze Zhang, Hao Zheng, Liang Kong*, Jiansheng Wu*, Dapeng Yu

I. UNIQUE IDENTIFIER OF TOPOLOGICAL ORDER

Topological orders can be characterized by their particle-like excitation, anyons. Anyon models describe the behavior of the topological sectors in a gapped system whose phase is subject to $(2+1)$d Topological Quantum Field Theory (TQFT). They can be described by unitary modular tensor categories (UMTC) mathematically [1]. We label the anyons in a finite set $\mathcal{C}$ by $a, b, c, \ldots$, and the vacuum sector by $\mathbf{1}$.

There are several major concepts in anyon models which we elaborate in formal mathematics in this section. The first is fusion between two anyons, where two anyons are combined together, or fused, to give an anyon. It is formulated by a fusion rule:

$$a \otimes b = \sum_c N_{ab}^c c, \tag{1}$$

the integer multiplicity N_{ab}^c gives the number of times c appears in the fusion outcomes of a and b. The quantum dimension d_a of an anyon a is defined through the fusion rule $d_a d_b = \sum_c N_{ab}^c d_c$. It represents the effective number of degrees of freedom of the anyon. For a non-Abelian anyon a, we have some b such that $\sum_c N_{ab}^c > 1$ and $d_a > 1$. If $\sum_c N_{ab}^c = 1$ for every b, then the anyon a is Abelian and we have $d_a = 1$. The integer N_{ab}^c is also the number of different ways in which a and b fuse to c hence is the dimension of the Hilbert space (the fusion space) $\hom(a \otimes b, c)$ where $\hom$ is the homomorphism. We choose an orthonormal basis of $\hom(a \otimes b, c)$, denoted diagrammatically by $\{$ 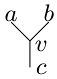 $\}_{v=1}^{N_{ab}^c}$.

Another important concept is braiding, which corresponds to the exchange of two anyons. The braiding operation of a and b in the fusion channel c is represented by

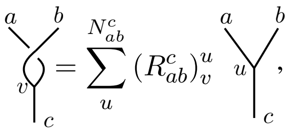

(2)

where R_{ab}^c is the so called R-matrix. If a or b is an Abelian anyon so that there exists a unique fusion channel $c = a \otimes b$, the matrix R_{ab}^c is reduced to a number R_{ab}. In particular, in an Abelian anyon model, as treated in this work, all the R-matrices can be organized into a single matrix $(R_{ab})_{a,b \in \mathcal{C}}$, which we also refer to as R-matrix by slightly abusing the terminology.

The two different ways of fusing three anyons a, b and c are related by the so called F-matrices:

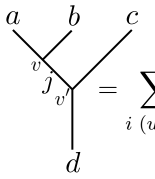

$$= \sum_{i\ (u,u')} \left(F^d_{abc}\right)^{i\ (uu')}_{j\ (vv')}$$

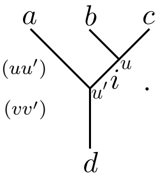

$$. \qquad (3)$$

In an Abelian anyon model, we have $d = a \otimes b \otimes c$ and the matrix F^d_{abc} is also reduced to a number F_{abc}. In the toric code model, it is easy to see that $F_{abc} \equiv 1$. We measure this value in the paper using scattering circuit with one additional ancilla control qubit to show that F-matrices are measurable in principle. However, for general anyon models, F-matrices can be nontrivial. For examples, the F-matrix of semions s is $F^s_{sss} = -1$ [2]. The F-matrices for non-Abelian anyons are even more complicated. For examples, the F-matrix for Fibonacci anyons τ is $F^\tau_{\tau\tau\tau} = \begin{pmatrix} \phi^{-1} & \phi^{-1/2} \\ \phi^{-1/2} & -\phi^{-1} \end{pmatrix}$ with $\phi = (1+\sqrt{5})/2$. The protocol we used in this article can be applied to measure the nontrivial F-matrices as well.

The above mentioned finite set $\mathcal{C}$, fusion rules N^c_{ab}, R- and F-matrices uniquely determine an anyon model.

In $(2+1)$d TQFT, anyons can have fractional spin and statistics. Rotating an anyon a by 2π (also called twisting) leads to a factor θ_a.

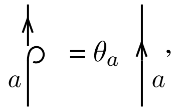

(4)

θ_a is called topological spin, relating to the ordinary angular momentum spin s_a by $\theta_a = e^{i2\pi s_a}$ and can be determined by R- and F-matrices through the equation

$$\theta_a = \frac{1}{d_a}$$

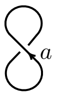

$$= \frac{R^1_{aa^*}}{d_a\ (F^a_{aa^*a})^1_1}. \qquad (5)$$

Indeed, the right-hand side of the equation involves a little bit of information about F-matrices in a subtle way. In an Abelian anyon model with trivial F-matrices, the equation is reduced to a rather simple one

$$\theta_a = R_{aa^*}. \qquad (6)$$

The effect of twisting (topological spin) is encoded in the modular T-matrix through the relation

$$T_{ab} = \theta_a \delta_{ab}. \qquad (7)$$

The fractional spins of anyons can be interpreted as their ribbon structure. Expressing anyons with ribbons implies the relation between twisting and braiding $(R^k)^c_{ab} = e^{k\pi i s_a} e^{k\pi i s_b} e^{-k\pi i s_c} I$ where I is the identity matrix of rank N^c_{ab}. In the case of $k = 2$, it gives the effect of double-braiding (moving a around b or moving b around a in a full-circle), where $(R^2)^c_{ab} = R^c_{ba} R^c_{ab} = e^{2\pi i s_a} e^{2\pi i s_b} e^{-2\pi i s_c} I = \frac{\theta_a \theta_b}{\theta_c} I$. The data of double-braiding is encoded in the modular S matrix

$$S_{ab} = \frac{1}{\mathcal{D}}$$
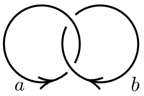

$$= \frac{1}{\mathcal{D}} \sum_c N^c_{ab} \frac{\theta_a \theta_b}{\theta_c} d_c, \quad (8)$$

where $\mathcal{D}$ is the total quantum dimension defined by $\mathcal{D} = \sqrt{\sum_a d_a^2}$. The S-matrix is determined by R-matrices through the relation

$$S_{ab} = \frac{1}{\mathcal{D}} \sum_c Tr(R^c_{ba} R^c_{ab}) d_c. \quad (9)$$

The fusion and braiding are related by the Verlinde formula

$$N^c_{ab} = \sum_d \frac{S_{ad} S_{bd} S^*_{cd}}{S_{1d}}. \quad (10)$$

The above-mentioned relations (7) and (9) indicate that the modular matrices S- and T-matrices can be deduced from R- and F-matrices. Since R- and F-matrices together can uniquely determine the topological order, we call them the unique identifier of topological order and provide a protocol to measure them.

II. ANYON CONDENSATION AND HALF BRAIDINGS ON GAPPED BOUNDARY

In this section, we elaborate on the mechanism of anyon condensation and half braidings in the context of the toric code model. Toric code model is a special case of quantum double model with group $\mathbb{Z}_2$ ($D(\mathbb{Z}_2)$). Mathematically, the anyons in QDM with group G can be labeled by pairs (C, π), where C is the conjugacy classes of G and π is the irreducible representations of the centralizers of C. The excitations on the boundary have a topological order given by a unitary fusion category (UFC). This fusion category is the representation category of a quasi-Hopf algebra and is Morita equivalent to the representation category $\mathrm{Rep}(G)$. The elementary excitations in the bulk are simple objects in the unitary modular tensor category (UMTC) $Z(\mathrm{Rep}(G))$, the Drinfeld center of $\mathrm{Rep}(G)$, which means the bulk is given by the boundary by taking Drinfeld center [3–5]. Suppose the boundary is described by a UFC $\mathcal{C}$. Then the bulk, the quantum double or Drinfeld center $Z(\mathcal{C})$ has objects labeled by pairs (x, e_x), where $x \in \mathcal{C}$ and e_x is a half braiding. The tensor product of the objects in the bulk is given by $(x, e_x) \otimes (y, e_y) = (xy, e_{xy})$, and the braiding is given by $c_{(x,e_x),(y,e_y)} = e_x(y)$. This mathematical structure sheds light on the characterization of a topological order through its boundary.

In our work, we experimentally measured the half braidings on the gapped boundary of toric code model. Further, the half braidings define the braidings (R matrices) in the bulk and other bulk properties (S and T matrices) can then be deduced.

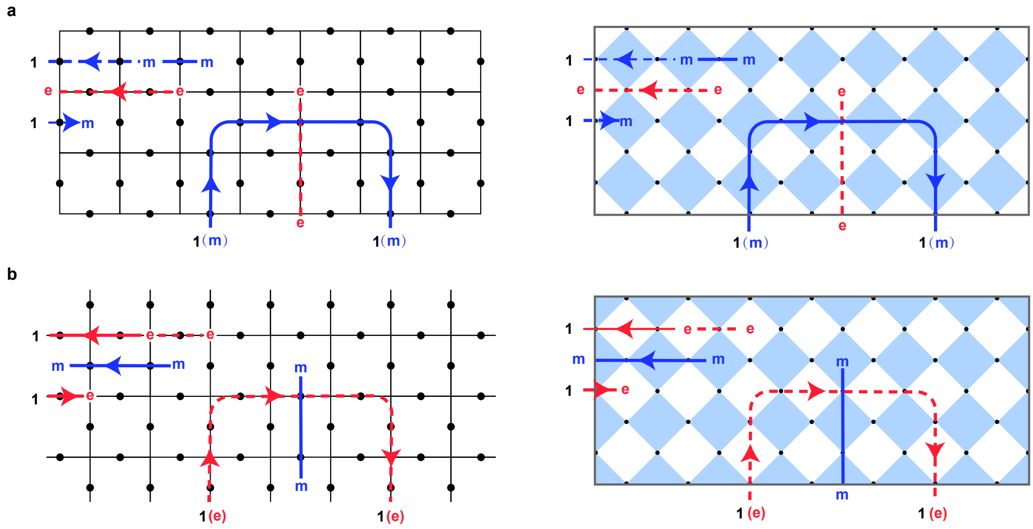

Figure S 1: Anyon condensation and half braiding on two types of boundaries of the toric code. a) Anyon condensation and half braiding on the smooth boundary (in the original vertex-plaquette form of toric code) and white boundary (in the plaquette form). b) Anyon condensation and half braiding on the rough boundary (in the original vertex-plaquette form of toric code) and blue boundary (in the plaquette form).

The bulk anyons for the toric code are $\{1, e, m, \varepsilon\}$. As illustrated in Fig. S1, there are two types of boundaries for the toric code: Type 1 (smooth or white) boundary and Type 2 (rough or blue) boundary. When an m anyon approaches a smooth (white) boundary, it condenses to vacuum, while ε condenses to e. The bulk to boundary map is $1, m \mapsto 1$, $e, \varepsilon \mapsto e$ and Type 1 boundary is also known as $\{1, e\}$ boundary according to its boundary excitations. This condensation procedure indicates that the m anyon can be created by a local operator acting on the boundary. But when m is moved into the bulk and become a bulk anyon, it was automatically endowed with additional structures called half braidings. The half braidings can be measured by moving this m around the boundary excitations 1 or e along a semi-circle near the boundary, which leads to a trivial or a -1 phase difference. This can be checked by applying all the vertex operators A_v surrounded by the path of m and the boundary (denoted as D) to the state $\prod_{v\in D} A_v |\Psi\rangle = \pm|\Psi\rangle$.

From these half braidings on the Type 1 boundary, we can then define braidings in the bulk and derive the R-matrix. The derivation of all the nontrivial braidings are listed in the following Table S i.

If we exam the Type 2, i.e. $\{1, m\}$ boundary, we can get another R-matrix (denoted by R'). Its nontrivial

Table S i: Nontrivial braidings of toric code model $D(\mathbb{Z}_2)$ defined by half braiding in type 1 boundary

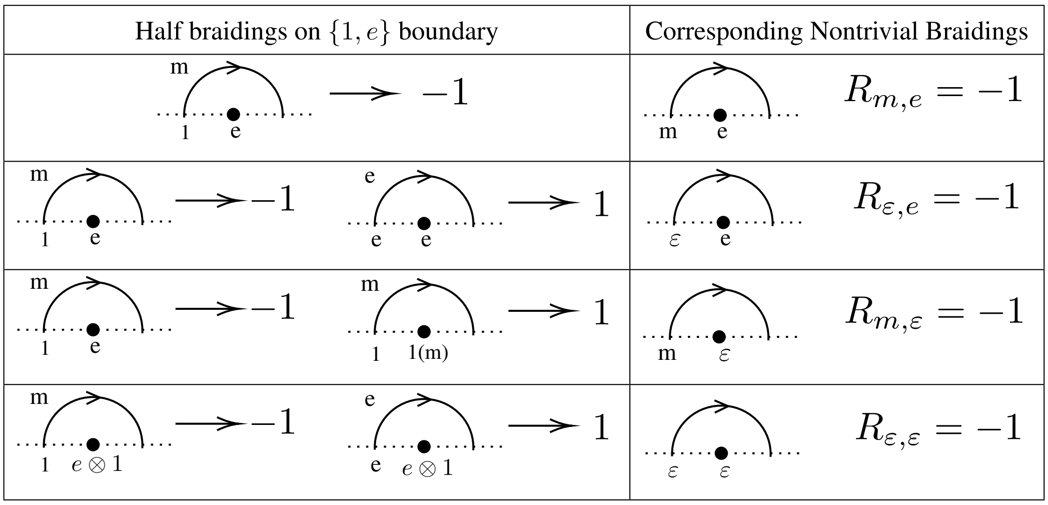

elements are listed in the following Table S ii.

Table S ii: Nontrivial braidings of toric code model $D(\mathbb{Z}_2)$ defined by half braiding in type 2 boundary

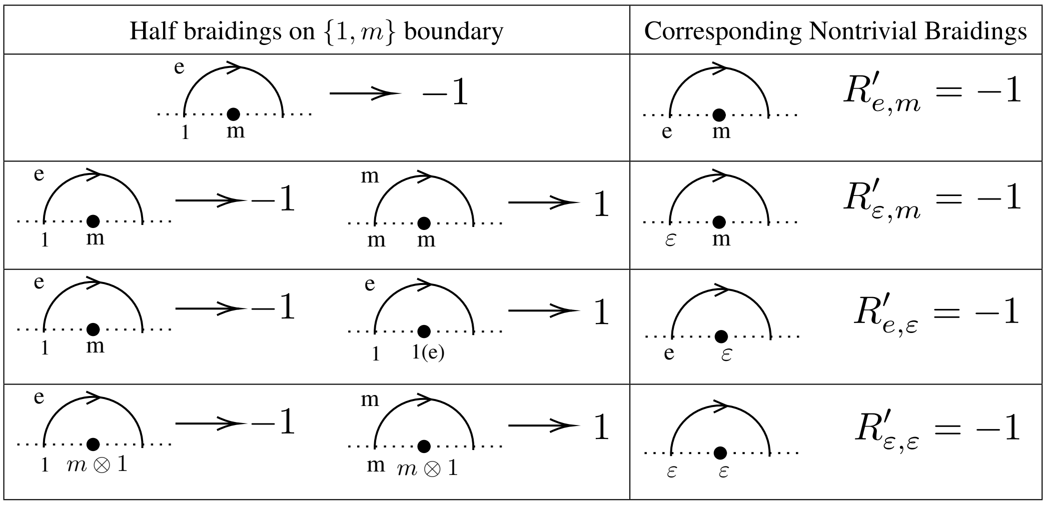

The following two R-matrices are defined by the half braidings on the two gapped boundaries of toric code lattice:

$$R = \begin{pmatrix} 1 & 1 & 1 & 1 \\ 1 & 1 & 1 & 1 \\ 1 & -1 & 1 & -1 \\ 1 & -1 & 1 & -1 \end{pmatrix}, \quad R' = \begin{pmatrix} 1 & 1 & 1 & 1 \\ 1 & 1 & -1 & -1 \\ 1 & 1 & 1 & 1 \\ 1 & 1 & -1 & -1 \end{pmatrix}. \tag{11}$$

The basis of the above matrices are $\{1, e, m, \varepsilon\}$. These two R-matrices describe all the braidings in the

bulk. Note that R and R' are equivalent in the sense of topological consistency equations and are related to each other by taking transpose (this is more clear in the second example $D(\mathbb{Z}_3)$). Each double-braiding (denoted as R^2) can be obtained by combining two braidings together, as illustrated in Table S iii, where two components of R are combined to get one element of R^2.

Table S iii: Nontrivial double braidings of toric code model $D(\mathbb{Z}_2)$

Braidings in the Bulk		Corresponding Nontrivial Double-braidings
m e $R_{m,e} = -1$	e m $R_{e,m} = 1$	m e $R^2_{m,e} = -1$
m ε $R_{m,\varepsilon} = -1$	ε m $R_{\varepsilon,m} = 1$	m ε $R^2_{m,\varepsilon} = -1$
e m $R_{e,m} = 1$	m e $R_{m,e} = -1$	e m $R^2_{e,m} = -1$
e ε $R^{e,\varepsilon} = 1$	ε e $R_{\varepsilon,e} = -1$	e ε $R^2_{e,\varepsilon} = -1$
ε e $R_{\varepsilon,e} = -1$	e ε $R_{e,\varepsilon} = 1$	ε e $R^2_{\varepsilon,e} = -1$
ε m $R_{\varepsilon,m} = 1$	m ε $R_{m,\varepsilon} = -1$	ε m $R^2_{\varepsilon,m} = -1$
ε ε $R_{\varepsilon,\varepsilon} = -1$	ε ε $R_{\varepsilon,\varepsilon} = -1$	ε ε $R^2_{\varepsilon,\varepsilon} = -1$

With the equations $\theta_a = R_{aa^*}$, $T_{ab} = \theta_a \delta_{ab}$ and $S_{ab} = \frac{1}{\mathcal{D}} R_{ba} R_{ab}$ discussed in the previous chapter, we can recover all the information about the bulk of the toric code model. For instance, we derive the S- and T- matrices:

$$S = \frac{1}{2}\begin{pmatrix} 1 & 1 & 1 & 1 \\ 1 & 1 & -1 & -1 \\ 1 & -1 & 1 & -1 \\ 1 & -1 & -1 & 1 \end{pmatrix}, \quad T = \begin{pmatrix} 1 & 0 & 0 & 0 \\ 0 & 1 & 0 & 0 \\ 0 & 0 & 1 & 0 \\ 0 & 0 & 0 & -1 \end{pmatrix}. \tag{12}$$

We can see different version of R-matrices (from different boundaries) give the same S- and T-matrices.

From the above results, we come to the conclusion that the bulk anyons are the boundary excitations

equipped with half braidings, which is physically measurable. Braidings in the bulk can be defined by the half braidings on one gapped boundary.

Our scheme can be straightforwardly applied to other Abelian anyon models. We take $D(\mathbb{Z}_3)$ as an illustrative example which also has trivial F-matrices. The method of deducing the bulk anyons and boundary excitations for quantum double models, in general, is discussed in [4, 6]. Here we will directly list the results. In terms of their charge and flux quantum numbers, the bulk anyons of $D(\mathbb{Z}_3)$ model are denoted as $\{1, e_1, e_2, m_1, m_2, e_1m_1, e_2m_1, e_1m_2, e_2m_2\}$. The boundary excitations on Type 1 and Type 2 boundaries are $\{1, e_1, e_2\}$ and $\{1, m_1, m_2\}$ respectively. The half braidings on the Type 1 boundary and the corresponding braidings are listed in Table S iv. It is worthwhile to point out that the half braidings between anyons in the two different classes (e-class $\{1, e_1, e_2\}$ and m-class $\{1, m_1, m_2\}$) and in the m-classes can be obtained through the picture of anyon condensation similar to the case of toric code discussed above. Indeed, two anyons inside the e-class or m-class have trivial mutual statistics, which is indicated by the Lagrangian subgroup principle [7–9]. The half braiding between anyons in e-class on $\{1, e_1, e_2\}$ boundary can be seen and measured through the following scheme (Fig. S2): Consider a smooth ($\{1, e_1, e_2\}$) boundary of a toric

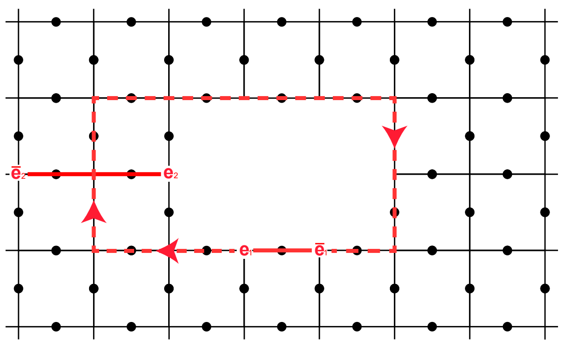

Figure S 2: Toric code on the torus with a smooth boundary.

code lattice on the torus where there is a boundary excitation e_2. The half braiding between e_1 and e_2 can be measured by first creating an anyon pair e_1-$\bar{e}_1$ on the boundary, then moving e_1 into the bulk along a semicircle around the boundary excitation e_2 and finally annihilating the anyon pair e_1-$\bar{e}_1$ on the boundary. The string operator of the full circle commutes with the string operator connecting e_2-$\bar{e}_2$ pair because they have no sharing qubit. Thus the full circle string is contractable and this circle operation has no effect on the quantum state. We can therefore conclude that the half braidings between e-class anyons equal to 1.

From the half braidings on the Type 1 ($\{1, e_1, e_2\}$) boundary and the corresponding braidings listed in

Table S iv, we obtain the following R-matrices in which $\omega = \exp(i2\pi/3)$ and $\overline{\omega} = \exp(-i2\pi/3)$,

$$R = \begin{pmatrix} 1 & 1 & 1 & 1 & 1 & 1 & 1 & 1 & 1 \\ 1 & 1 & 1 & 1 & 1 & 1 & 1 & 1 & 1 \\ 1 & 1 & 1 & 1 & 1 & 1 & 1 & 1 & 1 \\ 1 & \omega & \overline{\omega} & 1 & 1 & \omega & \overline{\omega} & \omega & \overline{\omega} \\ 1 & \overline{\omega} & \omega & 1 & 1 & \overline{\omega} & \omega & \overline{\omega} & \omega \\ 1 & \omega & \overline{\omega} & 1 & 1 & \omega & \overline{\omega} & \omega & \overline{\omega} \\ 1 & \omega & \overline{\omega} & 1 & 1 & \omega & \overline{\omega} & \omega & \overline{\omega} \\ 1 & \overline{\omega} & \omega & 1 & 1 & \overline{\omega} & \omega & \overline{\omega} & \omega \\ 1 & \overline{\omega} & \omega & 1 & 1 & \overline{\omega} & \omega & \overline{\omega} & \omega \end{pmatrix}, \quad R' = \begin{pmatrix} 1 & 1 & 1 & 1 & 1 & 1 & 1 & 1 & 1 \\ 1 & 1 & 1 & \omega & \overline{\omega} & \omega & \omega & \overline{\omega} & \overline{\omega} \\ 1 & 1 & 1 & \overline{\omega} & \omega & \overline{\omega} & \overline{\omega} & \omega & \omega \\ 1 & 1 & 1 & 1 & 1 & 1 & 1 & 1 & 1 \\ 1 & 1 & 1 & 1 & 1 & 1 & 1 & 1 & 1 \\ 1 & 1 & 1 & \omega & \overline{\omega} & \omega & \omega & \overline{\omega} & \overline{\omega} \\ 1 & 1 & 1 & \overline{\omega} & \omega & \overline{\omega} & \overline{\omega} & \omega & \omega \\ 1 & 1 & 1 & \omega & \overline{\omega} & \omega & \omega & \overline{\omega} & \overline{\omega} \\ 1 & 1 & 1 & \overline{\omega} & \omega & \overline{\omega} & \overline{\omega} & \omega & \omega \end{pmatrix}. \tag{13}$$

The modular matrices S- and T- can be obtained by using either of the two R-matrices,

$$S = \frac{1}{3}\begin{pmatrix} 1 & 1 & 1 & 1 & 1 & 1 & 1 & 1 & 1 \\ 1 & 1 & 1 & \omega & \overline{\omega} & \omega & \omega & \overline{\omega} & \overline{\omega} \\ 1 & 1 & 1 & \overline{\omega} & \omega & \overline{\omega} & \overline{\omega} & \omega & \omega \\ 1 & \omega & \overline{\omega} & 1 & 1 & \omega & \overline{\omega} & \omega & \overline{\omega} \\ 1 & \overline{\omega} & \omega & 1 & 1 & \overline{\omega} & \omega & \overline{\omega} & \omega \\ 1 & \omega & \overline{\omega} & \omega & \overline{\omega} & \overline{\omega} & 1 & 1 & \omega \\ 1 & \omega & \overline{\omega} & \overline{\omega} & \omega & 1 & \omega & \overline{\omega} & 1 \\ 1 & \overline{\omega} & \omega & \omega & \overline{\omega} & 1 & \overline{\omega} & \omega & 1 \\ 1 & \overline{\omega} & \omega & \overline{\omega} & \omega & \omega & 1 & 1 & \overline{\omega} \end{pmatrix}, \quad T = \begin{pmatrix} 1 & & & & & & & & \\ & 1 & & & & & & & \\ & & 1 & & & & & & \\ & & & 1 & & & & & \\ & & & & 1 & & & & \\ & & & & & \overline{\omega} & & & \\ & & & & & & \omega & & \\ & & & & & & & \omega & \\ & & & & & & & & \overline{\omega} \end{pmatrix}. \tag{14}$$

For the general Abelian quantum double model, note that the Abelian groups are either cyclic or direct products of cyclic groups, and the quantum double model of group $\mathbb{Z}_{d_1} \times \mathbb{Z}_{d_2}$ can be realized on a square lattice with two spins at each edge, one is d_1-level and the other is d_2-level. The anyons are just the combination of which $D(\mathbb{Z}_{d_1})$ and $D(\mathbb{Z}_{d_2})$ models. Our scheme can be straightly generalized to this case. Non-Abelian anyon models are more complicated and a general measurement protocol is proposed in the next section.

Table S iv: Half braidings of toric code model $D(\mathbb{Z}_3)$ defined on boundary and corresponding nontrivial braidings

Half braidings on $\{1, e_1, e_2\}$ boundary and Corresponding Nontrivial Braidings	
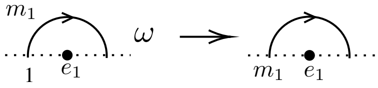 $R_{m_1,e_1} = \omega$	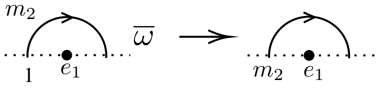 $R_{m_2,e_1} = \overline{\omega}$
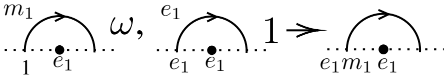 $R_{e_1m_1,e_1} = \omega$	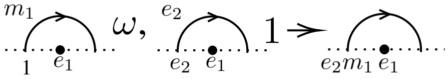 $R_{e_2m_1,e1} = \omega$
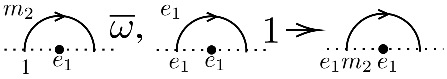 $R_{e_1m_2,e_1} = \overline{\omega}$	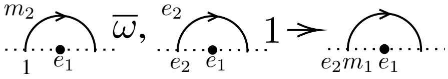 $R_{e_2m_2,e_1} = \overline{\omega}$
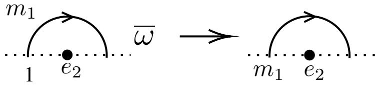 $R_{m_1,e_2} = \overline{\omega}$	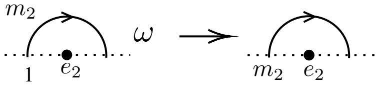 $R_{m_2,e_2} = \omega$
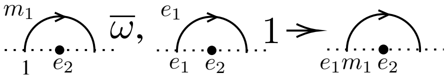 $R_{e_1m_1,e_2} = \overline{\omega}$	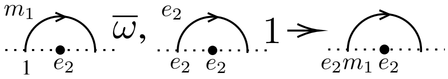 $R_{e_2m_1,e_2} = \overline{\omega}$
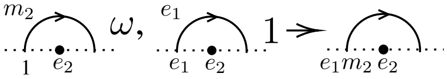 $R_{e_1m_2,e_2} = \omega$	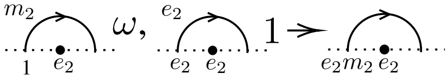 $R_{e_2m_2,e_2} = \omega$
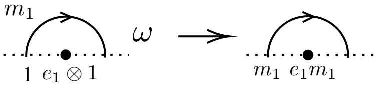 $R_{m_1,e_1m_1} = \omega$	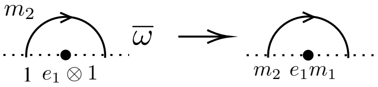 $R_{m_2,e_1m_1} = \overline{\omega}$
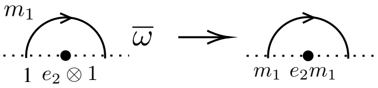 $R_{m_1,e_2m_1} = \overline{\omega}$	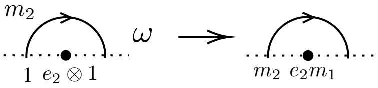 $R_{m_2,e_2m_1} = \omega$
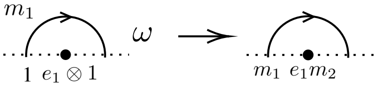 $R_{m_1,e_1m_2} = \omega$	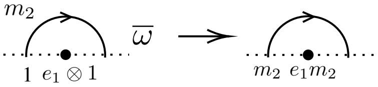 $R_{m_2,e_1m_2} = \overline{\omega}$
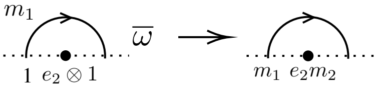 $R_{m_1,e_2m_2} = \overline{\omega}$	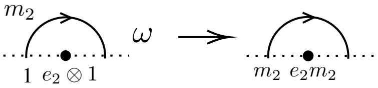 $R_{m_2,e_2m_2} = \omega$
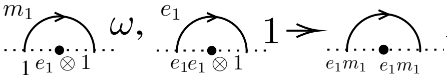 $R_{e_1m_1,e_1m_1} = \omega$	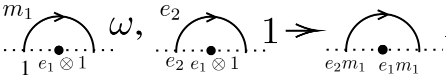 $R_{e_2m_1,e_1m_1} = \omega$
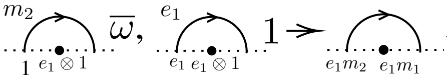 $R_{e_1m_2,e_1m_1} = \overline{\omega}$	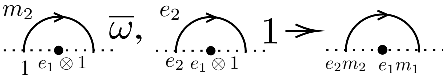 $R_{e_2m_2,e_1m_1} = \overline{\omega}$
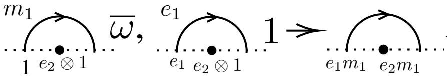 $R_{e_1m_1,e_2m_1} = \overline{\omega}$	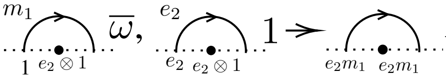 $R_{e_2m_1,e_2m_1} = \overline{\omega}$
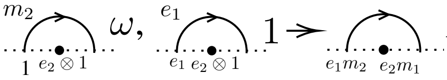 $R_{e_1m_2,e_2m_1} = \omega$	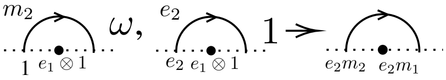 $R_{e_2m_2,e_2m_1} = \omega$
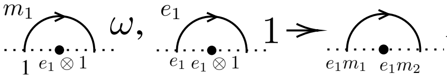 $R_{e_1m_1,e_1m_2} = \omega$	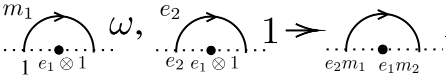 $R_{e_2m_1,e_1m_2} = \omega$
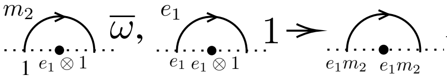 $R_{e_1m_2,e_1m_2} = \overline{\omega}$	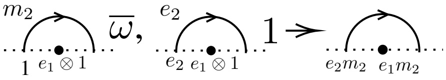 $R_{e_2m_2,e_1m_2} = \overline{\omega}$
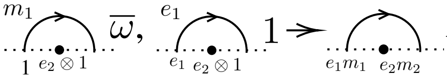 $R_{e_1m_1,e_2m_2} = \overline{\omega}$	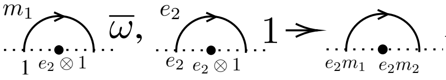 $R_{e_2m_1,e_2m_2} = \overline{\omega}$
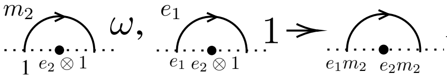 $R_{e_1m_2,e_2m_2} = \omega$	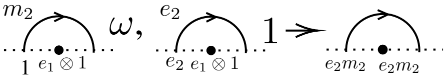 $R_{e_2m_2,e_2m_2} = \omega$

III. GENERAL MEASUREMENT SCHEME OF ANYON BRAIDING

Toric code model is a special case of the quantum double model (QDM) for group $\mathbb{Z}_2$. In this section, we propose a measurement protocol of anyon braiding (R-matrices) for QDM for an Abelian finite group G. For QDM, the bulk anyons form the UMTC $Z(\mathrm{Rep}(G))$, which is the Drinfeld center of $\mathrm{Rep}(G)$ [3, 4]. We can choose a convenient gapped boundary condition such that the boundary excitations form a unitary fusion category (UFC) $\mathcal{B}$ defined by $\mathrm{Rep}(G)$ (i.e. the category of finite dimensional G-representations).

When bulk anyons approach the boundary, they become boundary excitations and some bulk anyons are condensed to the vacuum on the boundary. These condensed anyons form a Lagrangian subgroup of G [7–9] or a Lagrangian algebra $\mathcal{A}$ in $Z(\mathrm{Rep}(G))$ [10]. Anyons in $a_i \in \mathcal{A}$ $(i = 1, ..., m)$ are bosons and have trivial mutual braidings

$$a_i \otimes a_j \xrightarrow{1} a_j \otimes a_i \xrightarrow{1} a_i \otimes a_j. \tag{15}$$

In the language of R-matrices, $R_{a_i,a_j} = 1$ for $i, j = 1, 2, ..., m$ (as illustrated in Fig. S3a).

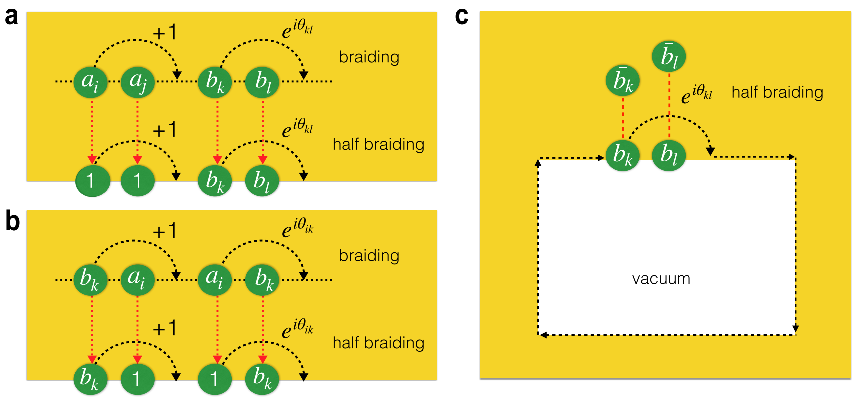

Figure S 3: **Braidings and half braidings in the quantum double model.** **a**, Anyons that condense to vacuum form a condensable algebra $\mathcal{A}$ and have trivial braidings. Anyons in UFC $\mathrm{Rep}(G)$ survived on the boundary and could have non-trivial braidings. **b**, $R_{b_k,a_i} = 1$ and $R_{a_i,b_k} = e^{i\theta_{ik}} \neq 1$ in general. **c**, Measurement of R_{b_k,b_l} through half braiding.

A generic boundary excitation $b_k \in \mathcal{B}$ $(k = 1, ..., n)$ can be realized by certain bulk anyons pushed to the boundary. By slightly abusing the notation, for each b_k, we choose a bulk anyon that becomes b_k when it approaches the boundary and still denoted it by b_k. Braidings between a_i and b_k can be obtained through half braidings illustrated in Fig. S3b. We have $R_{b_k,a_i} = 1$ $(i = 1, 2, ..., m; k = 1, 2, ..., n)$ because a_i is

the measured anyon which condenses to vacuum on the boundary and the circulation of anyon b_j along a semi-circle around a vacuum should not give any nontrivial phase factors. While $R_{a_i,b_k} = e^{i\theta_{ik}}$ $(i = 1, 2, ..., m; k = 1, 2, ..., n)$ is non-trivial in general since the moving anyon a_i is not vacuum any more after entering the bulk. The procedure for measuring R_{a_i,b_k} is summarized as follows: 1) Create anyon b_k (by string operators) on the boundary as the initial state; 2) Create anyon a_i on the boundary by local operators; 3) Move anyon a_i around anyon b_k along a semi-circle and annihilate a_i on the boundary and the resulting state works as the final state; 4) Compare the phase difference between the initial state and the final state.

Furthermore, the idea of measuring the half braidings between b_k and b_l is illustrated in Fig. S3c. First, we create two pairs of anyons b_k, $\bar{b}_k$ and b_l, $\bar{b}_l$ by string operators and push b_k, b_l to the boundary, where $\bar{b}_k$ and $\bar{b}_l$ are anti-particles of b_k and b_l, respectively. Then we half braid b_k with b_l and move b_k along a boundary back to its original position. In this way, the final state of the system goes back to the initial state with only a phase difference, which is R_{b_k,b_l}.

With all these measured R-matrices and the fact that anyons in bulk can be expressed as $(a_i \otimes b_k)$ $(i = 1, 2, ..., m; k = 1, 2, ..., n)$, all R-matrices for bulk anyons can be obtained via,

$$(a_i \otimes b_k) \otimes (a_j \otimes b_l) \xrightarrow{R_{a_i,b_l} R_{b_k,b_l}} (a_j \otimes b_l) \otimes (a_i \otimes b_k). \quad (16)$$

The measurement scheme proposed in this section can be further generalized to QDM for a non-Abelian group G or even to more general topological orders. However, in those more general situations, the braidings between two anyons, in general, are non-Abelian (i.e. not a phase any more). This difficulty can be overcome by performing the measurement of the half-braidings in a family of carefully prepared spaces of ground states with different sets of bulk anyons and boundary excitations. We leave the details and experimental implements to the future.

IV. THE SUBTLETY OF R-MATRIX MEASUREMENT

When preparing our manuscript, we noticed a recent theoretical microscopic construction of anyon data (R-matrices and F-matrices) [11], in which the authors used movement operators and splitting operators to construct the F-matrices and R-matrices.

However, we want to point out the subtlety of measuring the R-matrices in a microscopic lattice model as follows. Fig. S4 shows the graphic representation of the defining process of the R-matrices. In order to get states with two anyons a and b after splitting, the state before splitting can be (ab) and (ba) respectively.

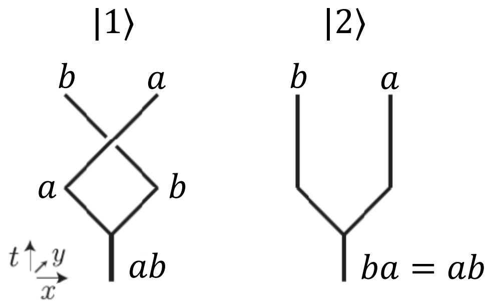

Figure S 4: Two graphic processes in which an anyon (ab) or (ba) splits into two anyons a and b. The final states $|1\rangle$ and $|2\rangle$ are related by the R-matrix $R_{a,b}$.

In a continuous model, (ab) and (ba) are all equal to c according to the fusion rule in the sense that a and b are in a region smaller than the correlation length and their relative positions don't matter. Therefore, the overlap of the two final states give rise to the R-matrix $\langle 2|1\rangle = R_{a,b}$. However, in a microscopic model on a lattice, this argument cannot be true. For example, in toric code model, e on the white plaquettes and m on the blue plaquettes form another kind of anyons $(em) = \varepsilon$, but their relative positions do still matter when calculating the overlap of wave functions. For the calculation of the article [11] to work, these two anyons should be exactly on the same lattice site.

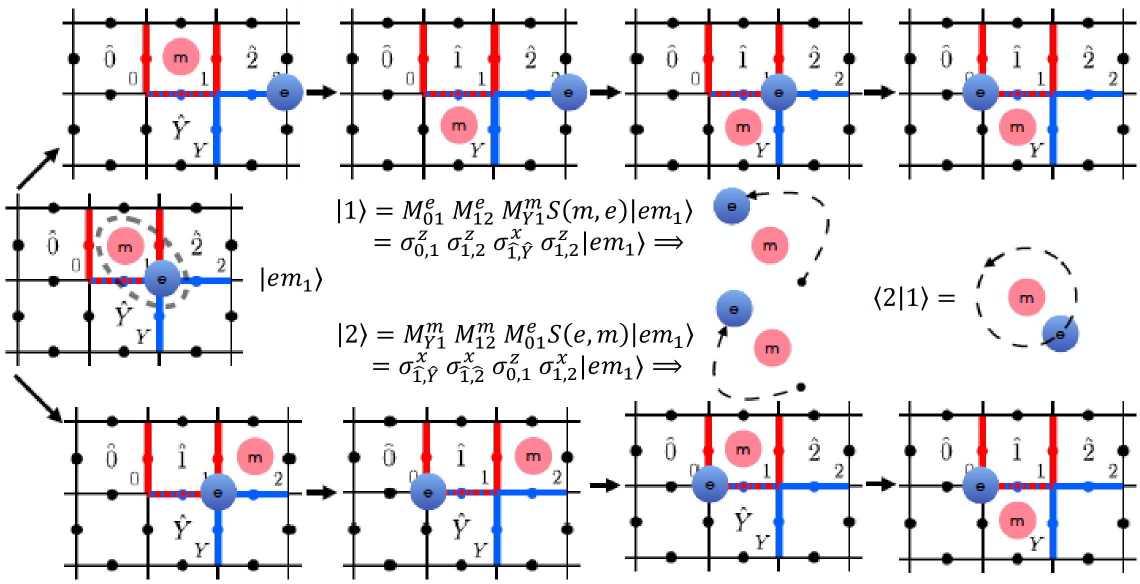

Figure S 5: The physical meaning of $|1\rangle$ and $|2\rangle$ states on a microscopic lattice model. $\langle 2|1\rangle$ is actually the mutual statistics (double-braiding), the S-matrix, instead of the R-matrix.

We elaborate this on their calculation of the claimed R-matrix of toric code model. In [11], the authors claimed that they did the following calculation involving splitting and moving operation of anyons shown

in Fig. S5 to get $R_{m,e} = \langle 2|1\rangle = -1$. We can see that from $|em_1\rangle$ to $|1\rangle$, e is effectively move around m counter-clockwise along a semi-circle, by comparing their relative positions in this process. From $|em_1\rangle$ to $|2\rangle$, e is effectively move around m clockwise along a semi-circle. Therefore, $\langle 2|1\rangle$ is the phase factor when e is moved around m counter-clockwise along a full-circle, which is double-braiding $S_{m,e}$ instead of braiding $R_{m,e}$.

From the above discussion, we can see the subtlety and difficulty in the calculation or measurement of the R-matrices. In fact, to calculate or measure the R-matrices, one has to overcome an obstacle that when moving a around b along a semi-circle (so-called braiding), the space configuration of the final state is different from that of the initial states. For examples, from intial state $|em_1\rangle$ to final state $|1\rangle$ is a braiding characterized by $R^{-1}_{e,m} = R_{e,m}$; From intial state $|em_1\rangle$ to final state $|2\rangle$ is a braiding characterized by $R_{m,e}$. We are able to overcome this difficulty by making use of bulk-boundary correspondence and anyon condensation for lattice models with small system sizes.

Furthermore, in Ref. [11], different versions of R-matrices and F-matrices are said to be due to different gauge choices. But in our paper, different versions of R-matrices result from choices of different boundary conditions. There are physical meanings for different versions of anyon data. In addition, we have demonstrated a key principle of topological order, i.e. the bulk-boundary correspondence.

V. EXPERIMENTAL DETAILS AND RESULTS

A. Quantum Processor

In our experiment, we perform the half braiding measurement and F-matrices measurement in the 3 and 4-qubit plaquette using a nuclear magnetic resonance (NMR) quantum simulator. All experiments are carried out on a Bruker Ascend NMR 600 MHz spectrometer at room temperature. The processor is a sample of ^{13}C-labeled trans-crotonic acid molecules dissolved in d$_6$-acetone. Their molecular structure and relevant parameters are shown in Fig. 3(f) of the main text. The sample consists of four ^{13}C 's labeled as C$_1$ to C$_4$ and they work as four qubits. The internal Hamiltonian of the system [12] is written as

$$\mathcal{H}_{\text{int}} = \sum_{j=1}^{4} \pi\nu_j\sigma_j^z + \sum_{4}^{j<k,=1} \frac{\pi}{2} J_{jk}\sigma_j^z\sigma_k^z, \tag{17}$$

where σ_j^z is the Pauli-Z operator of the j-th qubit, ν_j and J_{jk} are the chemical shifts and the J-coupling strengths between different qubits [13, 14], respectively.

B. Half Braiding Measurement: Interference and Tomography Approach

The half braiding of m, e anyons in toric code can be demonstrated in a plaquette surrounded by smooth boundaries consisting of three or four qubits (Fig. 3(a)(b) in the main text) with the following Hamiltonian,

$$\begin{aligned} H^{(3)} &= -\sigma_1^z\sigma_2^z\sigma_3^z - \sigma_1^x\sigma_2^x - \sigma_1^x\sigma_3^x - \sigma_2^x\sigma_3^x, \\ H^{(4)} &= -\sigma_1^z\sigma_2^z\sigma_3^z\sigma_4^z - \sigma_1^x\sigma_2^x - \sigma_2^x\sigma_3^x - \sigma_3^x\sigma_4^x - \sigma_1^x\sigma_4^x. \end{aligned} \tag{18}$$

They give the ground states

$$\begin{aligned} \left|\varphi_{\mathrm{g}}^{(3)}\right\rangle &= |000\rangle + |011\rangle + |101\rangle + |110\rangle\,, \\ \left|\varphi_{\mathrm{g}}^{(4)}\right\rangle &= |0000\rangle + |0011\rangle + |0101\rangle + |0110\rangle + |1001\rangle + |1010\rangle + |1100\rangle + |1111\rangle\,. \end{aligned} \tag{19}$$

A σ^z rotation of qubit 3 in the main text Fig. 3(a)(b) leads to a pair of e anyons in the vertexes near Q_3.

$$\begin{aligned} \left|\varphi_{\mathrm{e}}^{(3)}\right\rangle &= \sigma_3^z\left|\varphi_{\mathrm{g}}^{(3)}\right\rangle = |000\rangle - |011\rangle - |101\rangle + |110\rangle\,, \\ \left|\varphi_{\mathrm{e}}^{(4)}\right\rangle &= \sigma_3^z\left|\varphi_{\mathrm{g}}^{(4)}\right\rangle = |0000\rangle - |0011\rangle + |0101\rangle - |0110\rangle + |1001\rangle - |1010\rangle + |1100\rangle - |1111\rangle\,. \end{aligned} \tag{20}$$

We first prepare the superposition $|\varphi_{\mathrm{e}}\rangle + |\varphi_{\mathrm{e}}\rangle$ as initial state:

$$\begin{aligned} \left|\psi_{in}^{(3)}\right\rangle &= \left|\varphi_{\mathrm{g}}^{(3)}\right\rangle + \left|\varphi_{\mathrm{e}}^{(3)}\right\rangle = |000\rangle + |110\rangle\,, \\ |\psi_{in}^{(4)}\rangle &= \left|\varphi_{\mathrm{g}}^{(4)}\right\rangle + \left|\varphi_{\mathrm{e}}^{(4)}\right\rangle = |0000\rangle + |0101\rangle + |1001\rangle + |1100\rangle\,. \end{aligned} \tag{21}$$

The half braiding is achieved by moving a m anyon along Path 1, which leads to a relative phase:

$$\begin{aligned} \left|\psi_{fin-1}^{(3)}\right\rangle &= \sigma_3^x\sigma_1^x(\left|\varphi_{\mathrm{g}}^{(3)}\right\rangle + \left|\varphi_{\mathrm{e}}^{(3)}\right\rangle) = \left|\varphi_{\mathrm{g}}^{(3)}\right\rangle - \left|\varphi_{\mathrm{e}}^{(3)}\right\rangle = |101\rangle + |011\rangle\,, \\ \left|\psi_{fin-1}^{(4)}\right\rangle &= \sigma_3^x\sigma_4^x(\left|\varphi_{\mathrm{g}}^{(4)}\right\rangle + \left|\varphi_{\mathrm{e}}^{(4)}\right\rangle) = \left|\varphi_{\mathrm{g}}^{(4)}\right\rangle - \left|\varphi_{\mathrm{e}}^{(4)}\right\rangle = |0011\rangle + |0110\rangle + |1010\rangle + |1111\rangle\,. \end{aligned} \tag{22}$$

Another trivial Path 2 is taken for comparison. Moving a m anyon along Path 2 has no effect on the initial state:

$$\begin{aligned} \left|\psi_{fin-2}^{(3)}\right\rangle &= \sigma_2^x\sigma_1^x(|\varphi_{\mathrm{g}}\rangle + |\varphi_{\mathrm{e}}\rangle) = |\varphi_{\mathrm{g}}\rangle + |\varphi_{\mathrm{e}}\rangle = |000\rangle + |110\rangle\,, \\ \left|\psi_{fin-2}^{(4)}\right\rangle &= \sigma_2^x\sigma_4^x(|\varphi_{\mathrm{g}}'\rangle + |\varphi_{\mathrm{e}}'\rangle) = |\varphi_{\mathrm{g}}'\rangle + |\varphi_{\mathrm{e}}'\rangle = |0000\rangle + |0101\rangle + |1001\rangle + |1100\rangle\,. \end{aligned} \tag{23}$$

The final states after going through half braiding and trivial path can be differentiated through quantum state tomography. The experimental details and results is discussed in the following.

1. Experimental Process and Results

Our first experiment is divided into four steps: initialization of the processor, preparation of the superposition state, implementation of the half braiding process and measurement of the final state. We describe each part in detail in the following and the processes of the rest two experiments are similar.

1) *Initialization.* – As ensemble quantum computing, NMR qubits work at room temperature and the state of the NMR processor at thermal equilibrium is highly mixed [13]. So we need some "magic" to turn it into a pure state (say $|00...0\rangle$), which can serve as the initial state for quantum computing. The thermal equilibrium state of the 4-qubit NMR processor takes the form

$$\rho_{eq} = \frac{1-\epsilon}{16}\mathbb{I} + \epsilon \sum_{i=1}^{4} \sigma_i^z, \tag{24}$$

where $\epsilon = \hbar\omega_0/k_B T \approx 10^{-5}$ indicates the ratio of polarized spins and $\mathbb{I}$ is a 16×16 identity matrix. Note that the identity does not evolve under unitary propagator, and the only part that contributes to the NMR signal is the remaining term, which is called the *deviation density matrix*. Hence, we can simplify the analysis by focusing only on $\rho_{dev} \approx \sum_{i=1}^{4} \sigma_i^z$. Although ρ_{dev} is traceless and unnormalized, it indeed provides the same dynamics as well as much convenience compared with ρ_{eq} when studying NMR quantum computing.

Meanwhile, a pure state $|0000\rangle$ can be expanded in the Pauli basis as

$$\rho_{00} = |0000\rangle\langle 0000| = \left(\frac{I+\sigma_z}{2}\right)^{\otimes 4}. \tag{25}$$

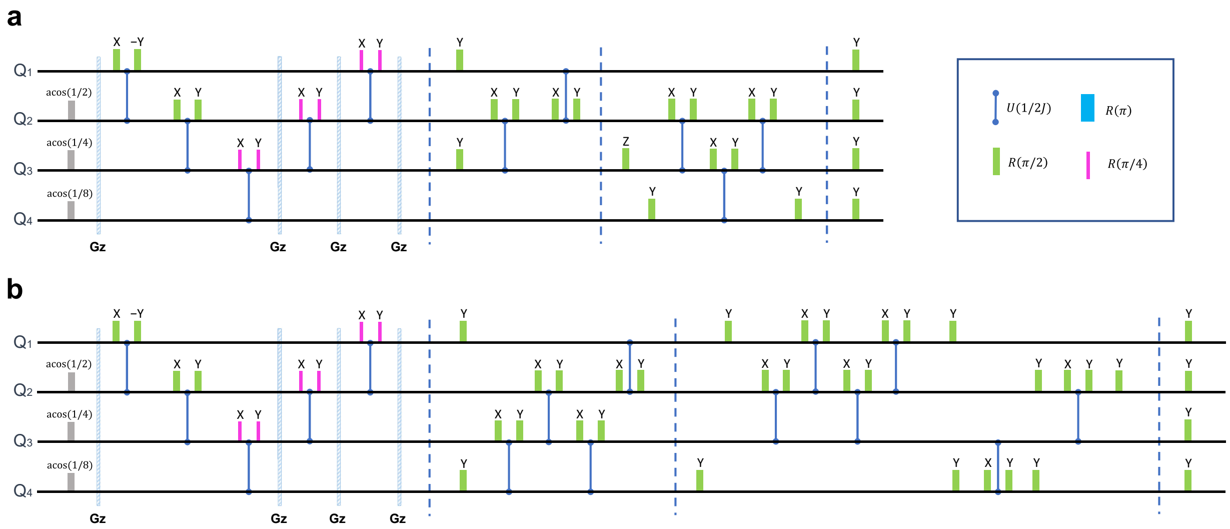

Figure S 6: Pulse sequence of the two scattering circuits. They are divided into four parts separated by dotted lines. (a) Pulse sequence of half braiding operation with a scattering circuit. The initialization step takes about 15 ms; the preparation of the ground state takes about 24.5 ms; the controlled-half braiding operations take out 27 ms. (b)Pulse sequence of fusion operation with a scattering circuit. The initialization step takes about 15 ms; the preparation of the ground state takes about 35 ms; the fusion operations take out 68.5 ms. All the pulses have theoretical fidelities over 0.99 in this experiment.

This expansion tells that ρ_{00} involves multiple orders of coherence except for the identity. The idea to create ρ_{00} from ρ_{dev} is to generate high-order coherence step by step while keeping their respective coefficients as required by Eq. (25). This process inevitably involves non-unitary operations due to the change of purity $trace(\rho^2)$ from ρ_{dev} to ρ_{00}.

The technique to prepare ρ_{00} in our experiment is called the spatial average technique [15]. The pulse sequences involved in this process can be seen on the left side of the first dashed lines in the circuits of Fig. S6. Other than regular single-qubit rotations, the initialization involves some $U(\frac{1}{2J})$ and Gz operators. $U(\frac{1}{2J})$ operation is used to increase the coherence order by one. For example, $U(\frac{1}{2J_{12}})$ between Q_1 and Q_2 means a unitary evolution under the coupling term $\frac{\pi}{2}J_{12}\sigma_1^z\sigma_2^z$ (see Eq. (17)) with duration $t = \frac{1}{2J_{12}}$. It is easy to verify that σ_1^x will be transformed to $\sigma_1^y\sigma_2^z$ by the evolution $U(\frac{1}{2J_{12}}) = e^{-i\frac{\pi}{4}\sigma_1^z\sigma_2^z}$, which is how the coherence order increases. Gz operator is the z-gradient field. It is a non-unitary operation to crush all terms in the transverse x-y plane and leave only the longitudinal term. Ideally, one can check that the sequence in Fig. S6 indeed creates ρ_{00} from ρ_{dev} up to an identity and a normalized factor.

Above is the "magic" in NMR quantum computing to initialize the processor. Since the created ρ_{00} is not genuinely pure due to the large omitted identity term, it is usually called a pseudo-pure state. In our experiment, the entire procedure of the pseudo-pure state creation takes around 15 ms with each operator realized by optimized pulses and refocusing schemes. We numerically simulated the fidelity and found it to be over 99.8%. In practice, we performed full state tomography after the initialization step and obtained fidelity beyond 99.8%.

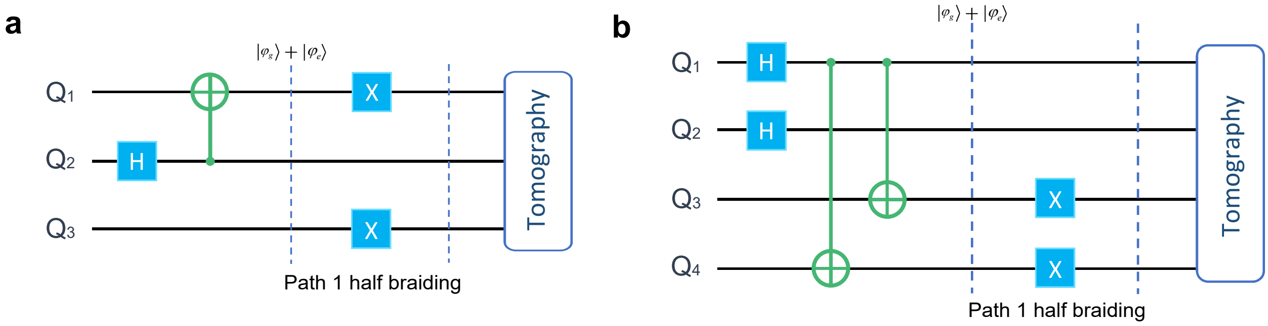

Figure S 7: Illustration of the experimental quantum circuits. The Hadamard and CNOT gates in the left of the first dashed lines are used to prepare the initial superposition state $|\psi_{in}^{(3)}\rangle = \left|\varphi_{\rm g}^{(3)}\right\rangle + \left|\varphi_{\rm e}^{(3)}\right\rangle$ and $|\psi_{in}^{(4)}\rangle = \left|\varphi_{\rm g}^{(4)}\right\rangle + \left|\varphi_{\rm e}^{(4)}\right\rangle$. These two circuits perform the nontrivial half braiding path (Path 1 in Fig. 3(a)(b) of the main text).

2) *Preparation of the superposition state and Implementation of the half braiding process.* – After initializing the processor, we can perform a standard quantum gate following the circuit diagram in Fig. S7. To reduce the gate errors in the experiment, all of the control pulses are obtained by the gradient ascent pulse

engineering (GRAPE) method and all the pulses have theoretical fidelities over 99.9%. First, the initial superposition state $|\psi_{\rm in}\rangle = |\varphi_{\rm g}\rangle + |\varphi_{\rm e}\rangle$ is prepared using a series of Hadamard and CNOT gates. They are realized by a single-qubit rotation $e^{-i\frac{\pi}{4}\sigma^y}$ and by exploiting the coupling between qubits, respectively. Considering CNOT gate between qubit 1 and 2 for the example, it can be realized by modifying the evolution operator $U_J(t) = e^{-i\frac{\pi}{2}J_{12}\sigma_1^z\sigma_2^z t}$ from the Hamiltonian (17) as

$$U_{CNOT} = \sqrt{i}Z_1\overline{Z_2}X_2U_J(1/2J)Y_2 = \begin{pmatrix} 1 & 0 & 0 & 0 \\ 0 & 1 & 0 & 0 \\ 0 & 0 & 0 & 1 \\ 0 & 0 & 1 & 0 \end{pmatrix}, \tag{26}$$

where Z_1, Z_2, X_2 and Y_2 stands for $e^{-i\frac{\pi}{4}\sigma_1^z}$, $e^{-i\frac{\pi}{4}\sigma_2^z}$, $e^{-i\frac{\pi}{4}\sigma_2^x}$ and $e^{-i\frac{\pi}{4}\sigma_2^y}$, respectively. Because of the varied coupling between different carbon nuclei, we optimize the circuit by taking carbon nuclei C_1, C_2, C_3 and C_4 as Q_4, Q_3, Q_2 and Q_1 in Fig. S7 respectively such that the CNOT gate can be implemented between carbon nuclei pairs (C_2-C_3, C_3-C_4) where the coupling is the largest.

Then we measure the prepared superposition states $|\psi_{in}\rangle$ for 3-qubit and 4-qubit experiments by full state tomography, obtaining the fidelity over 99% in numerical simulation and beyond 95.46% and 94.50% for 3 and 4-qubit experiment, respectively.

As discussed in the previous section, a half braiding (Path 1 in Fig. 3(a)(b)) will lead to a -1 phase factor for the excited state $|\varphi_{\rm e}\rangle$. It becomes a relative phase if we begin with the superposition $|\varphi_{\rm g}\rangle + |\varphi_{\rm e}\rangle$. As shown in Eq. (21)-(23), both half braiding path and the trivial path is implemented by two σ_x operator ($e^{-i\frac{\pi}{2}\sigma^x}$ rotation) on the corresponding qubits, leading to different final states. Then the initial superposition states, final states after half braiding path and final states after trivial path for 3 and 4-qubit experiment is measured through quantum state tomography.

3) *Measuring the final states via full state tomography.* – In the NMR system, only single-coherence operators can be directly observed. If we want to measure operators with higher coherence, additional readout pulses are required before we read the signal. Readout pulses will transfer the target operators to the single-coherence ones. In the 4-qubit quantum simulator, we can use 17 readout pulses to measure all the information of the density matrix:

$$\begin{aligned} &[XXXX, IIYY, YYXX, IIIY, XYXX,\\ &YXYI, IXYI, IIIX, XIYY, YXII, YYXY\\ &XYXI, IIYX, IXIY, IIXI, IYIY] \end{aligned} \tag{27}$$

The above process is the quantum state tomography(QST) [16]. In the NMR quantum simulation, QST is the most commonly used method to read out the signal. Here $X = e^{-i\frac{\pi}{4}\sigma^x}$, $Y = e^{-i\frac{\pi}{4}\sigma^y}$ and I is a

2×2 identity matrix. We can get all information about the final state with QST. Hence, we can estimate the quality of the experimental implementations by computing the fidelity between the theoretical final state ρ_{th} and the density matrix we get from the experiment.

We reconstruct the initial superposition state ρ_{in} and the final state ρ_{fin} after the half braiding and trivial braiding through Path 1 and Path 2 using a maximum likelihood approach [17] and the result is shown in Fig. 3 of the main text. We also calculate the fidelity between the numerical result and the experimental one. The average fidelities for Path 1 and Path 2 final states are 96.37% and 96.67% for the 3-qubit experiment and are 95.23% and 95.21% for the 4-qubit experiment, respectively.

C. General Half Braiding Measurement

In general, the effect of half braiding can be measured by the scattering circuit with one additional ancilla control qubit as proposed in [18], which is shown in Fig. S8(a).

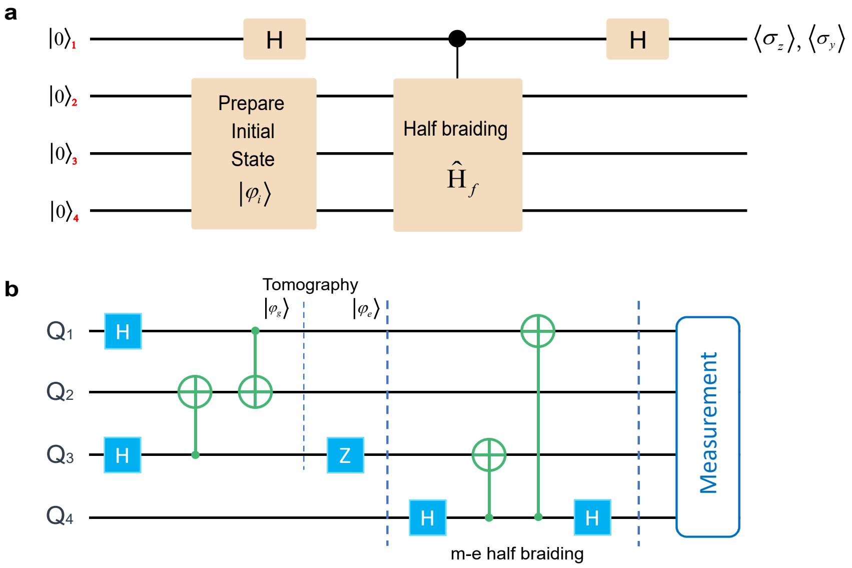

Figure S 8: (a) Scattering circuit for general measurement of half braidings. (b) Quantum circuit for measurement of m, e half braidings on 4-qubit quantum simulator. The expectation value $\langle\sigma^z\rangle$ is measured in this circuits.

In this circuit, the state before half braiding is prepared as the initial state $|\varphi_i\rangle$, in general, this circuit can be achieved via an adiabatic approach to prepare ground state (for a toric code Hamiltonian) following certain single-qubit gate on a quantum simulator. Then the half braiding is performed as a controlled operation. Finally, the expectation values $\langle\sigma^z\rangle$ and $\langle\sigma^y\rangle$ of the ancilla qubit is measured. One can get the

following results [18],

$$\langle\sigma^z\rangle = \mathrm{Re}[Tr(\hat{H}_f\,|\varphi_i\rangle\,\langle\varphi_i|)] = \mathrm{Re}(\langle\varphi_i|\,\hat{H}_f\,|\varphi_i\rangle),$$
$$\langle\sigma^y\rangle = \mathrm{Im}[Tr(\hat{H}_f\,|\varphi_i\rangle\,\langle\varphi_i|)] = \mathrm{Im}(\langle\varphi_i|\,\hat{H}_f\,|\varphi_i\rangle). \tag{28}$$

For Abelian anyon model, the half braiding ($\hat{H}_f$) leads to a phase factor, and can be obtained from the two expectation values $\langle\sigma^z\rangle$ and $\langle\sigma^y\rangle$ of the ancilla qubit.

In our experiment, we first prepare $Q1$-$Q3$ in Fig. S8(b) to the ground state of the 3-qubit toric code model $|\psi_0\rangle = |000\rangle + |011\rangle + |101\rangle + |110\rangle$ with fidelity 95.32%. Then a σ^z is applied to create two e anyons on the boundary. This excited state serves as the initial state in the scattering circuit. The half braiding is applied under the control of the ancilla qubit $Q4$ sandwiched by two Hadamard gates. $\langle\sigma_4^z\rangle$ and $\langle\sigma_4^y\rangle$ measures the real and imaginary parts of the overlap of the states before and after half braiding. We obtain $\langle\sigma_4^z\rangle = -0.930 \pm 0.004$ and $\langle\sigma_4^y\rangle = -0.081 \pm 0.003$ in the experiment. Finally, we normalize these two values such that their square sum to 1, calculate $\arctan\left(\frac{\langle\sigma_4^y\rangle}{\langle\sigma_4^z\rangle}\right)$ and obtain the phase angle $(1.027 \pm 0.001)\pi$, which is very close to the theoretical value π. Therefore, we confirm that the m-e half braiding on the smooth boundary leads to a -1 phase factor and then $R_{m,e} = -1$.

D. F-matrix measurement

As discussed in Section 1, different ways of fusing several anyons to get the same outcome (denoted by fusion trees) are different bases in the fusion Hilbert space. Changing between different bases is done by F move. Fig. 4(b) in the main text shows the two fusion trees for three toric code anyons together with their circle notations connected by F_{eem}^m, where ellipses enclose anyons. These ellipses are marked by the fusion outcome of the anyons enclosed.

For Abelian anyons, the fusion Hilbert space is of dimension 1, all the fusion trees are equivalent, and all elements of F are 1. The different trees, as shown in the case of Fig. 4(b) in the main text, are just different perspectives to interpret the same anyon configuration (e, e, m).

We verify this equation in our third experiment using a three-qubit plaquette with smooth boundary. The Hamiltonian and ground state is discussed in the previous section. As shown in Fig. 4(a) of the main text, in Path 1, we first create two e anyons by using σ_3^z, and an m anyon in the plaquette by applying σ_3^x. This corresponds to a circle notation and fusion diagram in Fig. 4(b)(i). In Path 2, we apply an operator σ_1^z to creat two e and then create an m anyon in the plaquette. Suppose the e anyon on the apex angle is far away from m and another e at the left base angle (this can be done by creating two e anyons by a long string operator in a larger toric code lattice), then the m anyon is simultaneously fused with the e anyon at the left

base angle once it is created. Then we apply another string operator σ_2^z to move the apex e to the right base angle. Thus the circle notation and fusion diagram in Fig. 4(b)(ii) is realized.

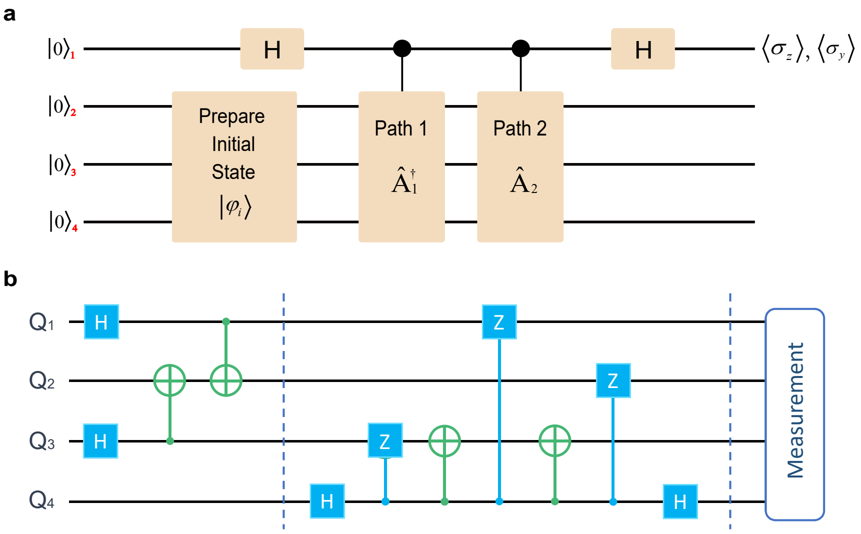

Figure S 9: (a).Scattering circuit for the measurement of F_{eem}^m, where $A_1 = \sigma_3^x\sigma_3^z$, $A_2 = \sigma_2^z\sigma_3^x\sigma_1^z$. (b).Quantum circuit measuring F_{eem}^m on 4-qubit NMR quantum simulator, where Q_4 is the control qubit. The expectation value $\langle\sigma_4^z\rangle$ is measured in this circuits.

The measurement of F-matrix is achieved by the scattering circuits shown in Fig. S9, $Q1$-$Q3$ are first prepared to the ground state of the 3-qubit toric code model $|\psi_0\rangle = |000\rangle + |011\rangle + |101\rangle + |110\rangle$ as initial state. Between two Hadamard gates on the ancilla qubit Q_4, two approaches to reach the same anyon configuration described above and shown in Fig. 4(a) are applied under the control of Q_4. Finally, $\langle\sigma_4^z\rangle$ and $\langle\sigma_4^y\rangle$ are measured to compare the two final states.

In this experiment, we choose C_3 in the molecule to be the control qubit to reduce the complexity of the circuit. C_1, C_2 and C_4 in the molecule represent Q_1, Q_2, Q_3 in the circuit (Fig. S9(b)), respectively. The ground state of the 3-qubit toric code model $|\psi_0\rangle = |000\rangle + |011\rangle + |101\rangle + |110\rangle$ is prepared with fidelity 92.96% on $Q_1 - Q_3$. Then the operator $A_1^\dagger = (\sigma_3^x\sigma_3^z)^\dagger$ and $A_2 = \sigma_2^z\sigma_3^x\sigma_1^z$ corresponding to Path 1 and Path 2 in Fig. 4(a) is applied to the three toric code qubits under the control of $Q4$ between two Hadamard gates. We measure $\langle\sigma_4^z\rangle$ and $\langle\sigma_4^y\rangle$ of the control qubit to compare the final states of the two paths and obtain $\langle\sigma_4^z\rangle = 0.712 \pm 0.006$ and $\langle\sigma_4^y\rangle = 0.177 \pm 0.004$ in the experiment. We normalize them such that they square sum to 1 and obtain the angle $\arctan\left(\frac{\langle\sigma_4^y\rangle}{\langle\sigma_4^z\rangle}\right) = (0.077 \pm 0.002)\pi$. Theoretically, $\langle\sigma_4^z\rangle = 1$, $\langle\sigma_4^y\rangle = 0$ and $\arctan\left(\frac{\langle\sigma_4^y\rangle}{\langle\sigma_4^z\rangle}\right) = 0$. Therefore, we verified $F_{eem}^m = 1$ in this experiment.

[1] Kitaev, A. Anyons in an exactly solved model and beyond. *Ann. Phys.* **321**, 2–111 (2006).

[2] Rowell, E., Stong, R. & Wang, Z. On Classification of Modular Tensor Categories. *Comm. Math. Phys.* **292**, 343–389 (2009).

[3] Kitaev, A. & Kong, L. Models for gapped boundaries and domain walls. *Comm. Math. Phys.* **313**, 351 (2012).

[4] Cong, I., Cheng, M. & Wang, Z. Topological quantum computation with gapped boundaries. *Preprint at https:// arXiv:1609.02037* (2016).

[5] Kong, L., Wen, X.-G. & Zheng, H. Boundary-bulk relation in topological orders. *Nucl. Phys. B* **922**, 62–76 (2017).

[6] Kómár, A. *Quantum Computation and Information Storage in Quantum Double Models*. Thesis (2018).

[7] Kapustin, A. & Saulina, N. Topological boundary conditions in abelian Chern–Simons theory. *Nucl. Phys. B* **845**, 393–435 (2011).

[8] Levin, M. Protected edge modes without symmetry. *Phys. Rev. X* **3**, 021009 (2013).

[9] Barkeshli, M., Jian, C.-M. & Qi, X.-L. Classification of topological defects in Abelian topological states. *Phys. Rev. B* **88**, 241103 (2013).

[10] Kong, L. Anyon condensation and tensor categories. *Nucl. Phys. B* **886**, 436 – 482 (2014).

[11] Kawagoe, K. & Levin, M. Microscopic definitions of anyon data. *Phys. Rev. B* **101**, 115113 (2020).

[12] Vandersypen, L. M. & Chuang, I. L. NMR techniques for quantum control and computation. *Rev. Mod. Phys.* **76**, 1037 (2005).

[13] Li, K. *et al.* Experimental identification of non-abelian topological orders on a quantum simulator. *Phys. Rev. Lett.* **118**, 080502 (2017).

[14] Xin, T. *et al.* Local-measurement-based quantum state tomography via neural networks. *npj Quantum Information* **5**, 1–8 (2019).

[15] Cory, D. G., Fahmy, A. F. & Ha, T. F. Ensemble quantum computing by NMR spectroscopy. *PNAS* **94**, 1634–1639 (1997).

[16] Xin, T., Pedernales, J. S., Solano, E. & Long, G.-L. Entanglement measures in embedding quantum simulators with nuclear spins. *Phys. Rev. A* **97**, 022322 (2018).

[17] Hradil, Z., Rehácek, J., Fiurasek, J. & Jezek, M. *Quantum State Estimation Ch.3* (Springer-Verlag, Berlin Heidelberg, 1999).

[18] Miquel, C. *et al.* Interpretation of tomography and spectroscopy as dual forms of quantum computation. *Nature* **418**, 59 (2002).
